# Cochlear impulse responses resolved into sets of gammatones: the case for beating of closely spaced local resonances

**DOI:** 10.7717/peerj.6016

**Published:** 2018-11-27

**Authors:** Andrew Bell, Hero P. Wit

**Affiliations:** 1 John Curtin School of Medical Research, Australian National University, Canberra, ACT, Australia; 2 Department of Otorhinolaryngology/Head and Neck Surgery, University of Groningen, Groningen, Netherlands

**Keywords:** Impulse response, Coupled oscillators, Gammatones, Beating, Instantaneous frequency, Basilar membrane, Tectorial membrane

## Abstract

Gammatones have had a long history in auditory studies, and recent theoretical work suggests they may play an important role in cochlear mechanics as well. Following this lead, the present paper takes five examples of basilar membrane impulse responses and uses a curve-fitting algorithm to decompose them into a number of discrete gammatones. The limits of this ‘sum of gammatones’ (SOG) method to accurately represent the impulse response waveforms were tested and it was found that at least two and up to six gammatones could be isolated from each example. Their frequencies were stable and largely independent of stimulus parameters. The gammatones typically formed a regular series in which the frequency ratio between successive members was about 1.1. Adding together the first few gammatones in a set produced beating-like waveforms which mimicked waxing and waning, and the instantaneous frequencies of the waveforms were also well reproduced, providing an explanation for frequency glides. Consideration was also given to the impulse response of a pair of elastically coupled masses—the basis of two-degree-of-freedom models comprised of coupled basilar and tectorial membranes—and the resulting waveform was similar to a pair of beating gammatones, perhaps explaining why the SOG method seems to work well in describing cochlear impulse responses. A major limitation of the SOG method is that it cannot distinguish a waveform resulting from an actual physical resonance from one derived from overfitting, but taken together the method points to the presence of a series of closely spaced local resonances in the cochlea.

## Introduction

Gammatones have had a long history in auditory studies (Lopez-Najera, Lopez-Poveda & Meddis, 2007; Lopez-Poveda & Meddis, 2001; Lyon, 2017; Lyon, Katsiamis & Drakakis, 2010; Patterson et al., 1992) and in electronic engineering (Ngamkham et al., 2010; Tucker, 1946), but their direct application to cochlear mechanics has been more limited. The survey of cochlear models by Lyon (2017) is useful in showing a strong link between gammatones and cochlear models. As Lyon notes, gammatones, or something very close to them, are evident in nearly all systems used in modelling cochlear filter shapes (p. 161, referring to Papoulis (1962), pp. 234–236). Of particular interest, the whole gammatone family is characterised by the property of having multiple coincident poles (Lyon, 2017, Ch. 9). Lyon proceeds to show that when there are coincident poles (and coincident natural frequencies) the system will have an impulse response which resembles a gammatone.

Here, we explore this property. Essentially, if cochlear impulse responses derive from a system of coincident poles—as in a two-degree-of-freedom (2-DOF) model—then they should be made up of a number of gammatones. In reverse, it might be possible to fit a series of constant-frequency gammatones to the impulse responses, and this is what is attempted here using a numerical fitting algorithm we call the ‘sum of gammatones’ (SOG) method.

Elliott, Ni & Sun (2017) fitted data from the Ogahalai lab—velocity and phase of the mouse cochlea in response to sinusoidal stimuli of 10, 30, 50, and 70 dB SPL (Lee et al., 2015)—with a ‘coupled-box’ model of the mammalian cochlea which contained 2-DOF micromechanical elements. The micromechanical elements were coupled by fluid according to that in Elliott, Lineton & Ni (2011), and the general form of the basilar membrane (BM) admittance of the coupled-box model had four poles and three zeros. The impulse response of the fitted coupled-box model was calculated by inverse Fourier transform, and it is of interest to note that, despite the complexity of what was happening inside the box, the calculated impulse response broadly resembled a gammatone—oscillations of constant frequency which rose and then slowly decayed. An examination of how closely this impulse response could be approximated by the sum of a small number of gammatones of closely matched frequencies is one of the case studies in our work (Case 6 below).

**Figure 1:**
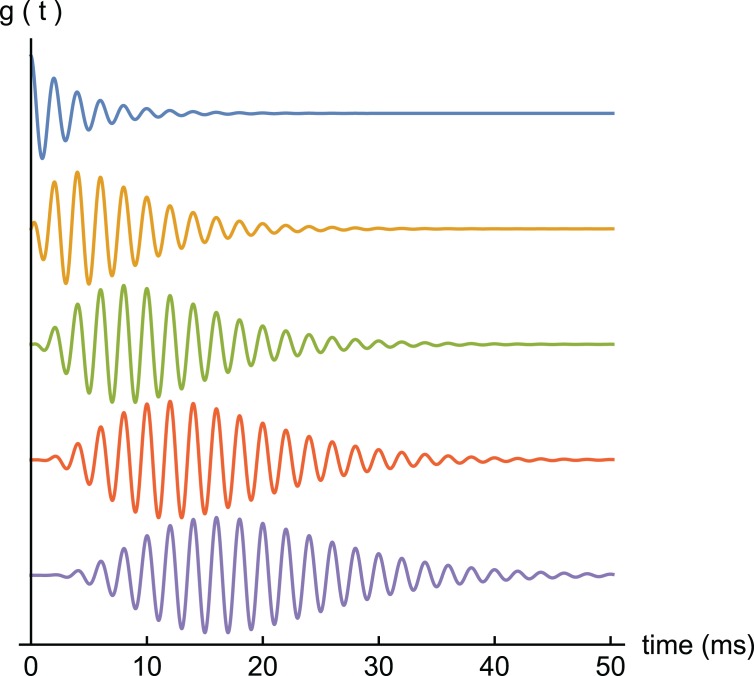
Gammatone profiles. A set of gammatones of increasing order (*n* = 1, top, to *n* = 5, bottom). In all examples the frequency is 0.5 kHz and decay factor *b* = 0.25.

Because gammatones have a single frequency, they are easy to analyse and implement. They approximate a cascade of resonant filters (Tucker, 1946) (see also Supplementary Material S1 and p. 177 of Lyon (2017)) and, depending on parameters, satisfy a variety of frequency response shapes (Ch. 9 of Lyon (2017)). Figure 1 shows examples of gammatones of order 1 to 5, and it is noteworthy that, despite certain differences, the envelopes of the higher order ones resemble cochlear impulse responses. Tucker’s result is of interest in showing that if a resonant filter (or harmonic oscillator) is driven by an impulse, the result will be a gammatone. More generally, Tucker demonstrated that in a cascade of such filters, if the input to one stage is a gammatone of order *n*, its output will be a gammatone of order *n* + 1. This result forms part of Supplementary Material S2.

The gammatone function of order *n* is given by:

(1) $$g\left(t \right){\rm{ }} = {t^n}^{-1}{\rm{exp}}\left({-bt} \right){\rm{ cos}}\left({{\rm{\omega }}t + \rm\phi } \right)$$

where *t* is time, *b* is decay rate, ω is angular frequency, *n* is 1, 2, 3,…, and φ is phase. Gammatones are therefore sine waves of fixed frequency with envelope *t*^*n*−1^ exp(−*bt*). For *b* = 1, the definite integral from zero to infinity of this envelope is the gamma function, Γ(*n*), hence the name. Numerically, Γ(*n*) is just (*n*–1)!.

The above theoretical perspectives encouraged us to gather cochlear impulse responses and test how well they could be fitted using the SOG method.

### Impulse responses

Any linear time-invariant mechanical or electrical system can be uniquely described by its impulse response, the waveform produced following a brief impulse—in auditory terms a click.

Experiments have shown that impulse responses of the BM show the following characteristics (elaborated from Shera (2001b)). (1) They display many cycles, meaning that the BM has a narrow-band frequency response and high *Q*. (2) There is gradual onset and decay. (3) The waveform typically shows a rising instantaneous frequency (IF) during onset, usually an upwards sweep or frequency glide which begins below the characteristic frequency (CF) and converges towards it. (4) As sound levels increase, zero crossings of the waveform remain fixed in time. (5) The spectrum of the impulse response quite often shows multiple peaks, typically at a ratio near 1.1. (6) If the response is sufficiently long, a series of lobes is often apparent in which the envelope goes through cyclic waxing and waning. (7) Where a second lobe exists, it typically contains about 10 cycles, irrespective of the CF, and the waveform within it is often 180° out of phase with that in adjacent lobes.

Early investigators made use of nerve fibre recordings (De Boer & Kruidenier, 1990; Møller & Nilsson, 1979) because they are easier to acquire than direct observations of the BM. Nerve fibre recordings provide an indirect view of the cochlea and its filtering properties, and much can be learnt by treating the recordings as rectified and time-delayed versions of the BM motion. An electrode recording can be reverse correlated with the sound input, and the resulting reverse correlation (revcor) function can provide an estimate of the impulse response of the matching local filter. The drawback is that the intervening neural transduction process must be inferred, and this can be problematic. Direct observations, as used in the cases we analyse here, are more revealing, providing detailed information on the dynamics of the cochlear filters.

In general, we find that most cochlear impulse responses can be well fitted by gammatones, of order 3 or 4, both in the time and frequency domains. The analysis also reveals that the gammatones are usually regularly spaced at a frequency ratio of about 1.05–1.10. A possible interpretation, presented in more detail later, is that the pairs of closely spaced poles might have a connection with pairs of oscillating elements—perhaps (as assumed in a typical 2-DOF model) the BM and the tectorial membrane (TM). If these elements are elastically coupled, they mutually exchange energy and produce a beating-like waveform when excited. In fact, we show here that the impulse response of a coupled oscillator system gives rise to a waveform that can be well approximated by the sum of two second-order gammatones, suggesting that the waxing and waning evident in cochlear impulse responses may involve the beating behaviour of two component masses—possibly the BM and the TM.

It needs to be kept in mind, of course, that the cochlea is not just a system of lumped micromechanical elements but a distributed system (Lyon, 2017, Ch. 12), so it is difficult to see how an extended series of such elements, coupled together, could preserve discrete features in the impulse response. Nevertheless, lumped parameter models might provide a suitable starting point (see Ni et al. (2014) for a review), and it could be significant that the impulse response of an oscillator located within a chain of similar oscillators—as in the classic vibrating reed frequency meter—can exhibit waxing and waning (Bell & Wit, 2015; Fig. 14A). The SOG method suggests that multiple gammatone-like waveforms do seem to be present in the cochlea, at least at the observation sites where impulse responses were recorded. Speculatively, later discussion links the gammatones to quantised frequency steps that have already been identified in the cochlea.

Finally, our analysis focuses on the instantaneous frequencies (IFs) of the impulse responses, which were examined by Hilbert transform. This work reveals a characteristic pattern of upwards surges (or glides) in the impulse responses. Significantly, it is shown that when two constant-frequency gammatones beat together, they also produce upwards (or downwards) surges in IF, suggesting it is possible that the glides observed in the cochlea may have appreciable contributions from the beating of underlying gammatones, or in terms of the basic 2-DOF model, physical interaction between the BM and TM.

This work is exploratory in nature, and many questions remain open. The mechanics of the actual cochlea is more complicated than that of two coupled masses, but nevertheless, by taking the simplest case as a starting point and seeing how far it can be taken, it appears as if some quantised frequency features are preserved in recorded impulse responses.

## Methods

Numerical data of experimental cochlear impulse responses published in the literature were kindly provided by the authors. The waveforms were decomposed into gammatones using the FindFit procedure in Mathematica 11 (Wolfram Research, Champaign, IL, USA) and using Abscissa 3.4.2 (http://rbruehl.macbay.de). Explicitly, the software was instructed to fit the sum of two or more gammatones of the form given in Eq. (1) to the data.

A key property of a gammatone is that it has constant frequency over time—that is, its IF is invariant. For a review of gammatones and their applicability in auditory studies, see Lyon, Katsiamis & Drakakis (2010) and Lyon (2017).

In total, some dozens of impulse responses from eight or more authors were analysed, covering a wide range of animals and techniques. In some instances, impulse responses from theoretical cochlear models were also available, and these provided useful confirmation. This paper presents five informative examples, each as a separate case.

Each fit to a gammatone (Eq. (1)) carries five free parameters, and for two gammatones of identical order, there are nine. To minimise the number of free parameters, the order, *n*, was fixed at *n* = 3 or 4, which appeared to best match the profile of most cochlear impulse responses. To quickly find starting values for a fit to the more complex waveforms, the approach was to first use Abscissa to fit the tail of the impulse response (called the coda by Li & Grosh (2016)), where the dynamics is simpler, involving only the long-lasting responses. Then, having satisfactorily identified these terms, they were subtracted from the total waveform and a search made for additional gammatones in the residual (the earlier parts of the waveform). The criterion for a good fit, determining the total number of gammatones necessary, was that no substantial difference could be observed between the response and the fit, and in practice this meant that the RMS amplitude of the residual was less than 5% of the original signal. Examples of the difference between the response and the fit, in both the time and frequency domains, are shown in the Results; a step-by-step Mathematica notebook showing how the fits are made is provided as a Supplementary File.

Usually only a small number of gammatones (two to four) were sufficient to produce a good fit, but sometimes up to six were needed. The constancy of the extracted gammatone frequencies, despite different stimulus intensities and other conditions, was taken as an indication that the retrieved gammatones were an innate property of the data and not generated by the fitting (with a range of free parameters it would be unlikely for the frequency to stay constant if the retrieved gammatone were an artefact). However, since any arbitrary waveform can be decomposed into gammatone wavelets (Adiga, Magimai-Doss & Seelamantula, 2013)—just like the Fourier transform decomposes any waveform into sine waves—what other evidence is there that the components extracted by numerical decomposition are ‘real’? This is an important issue addressed in the ‘Discussion’, where it is suggested that at least the first few recovered gammatones may reflect some actual cochlear oscillations, although more work will be needed to gauge how far the fitting process can be pushed.

The IF is another important characteristic of cochlear impulse responses, and this quantity was also investigated, both for the original waveform and for waveforms reconstructed from identified gammatones. The IF was computed by Hilbert transform, and sometimes also by calculating the time between zero-crossings.

## Results

In general, all impulse responses examined could be well fitted by a small number of gammatones. For simple single-burst waveforms derived from low sound pressure levels (<60 dB SPL), two or three gammatones were sufficient, but several more were required for higher intensity conditions. Most of the impulse responses showed waxing and waning, and in these cases the multiple lobes could always be explained in terms of the beating of the component gammatones. The implications of this basic finding are detailed in the ‘Discussion’, where the case is made that the results support a beating model of some kind.

### Case 1

**Figure 2:**
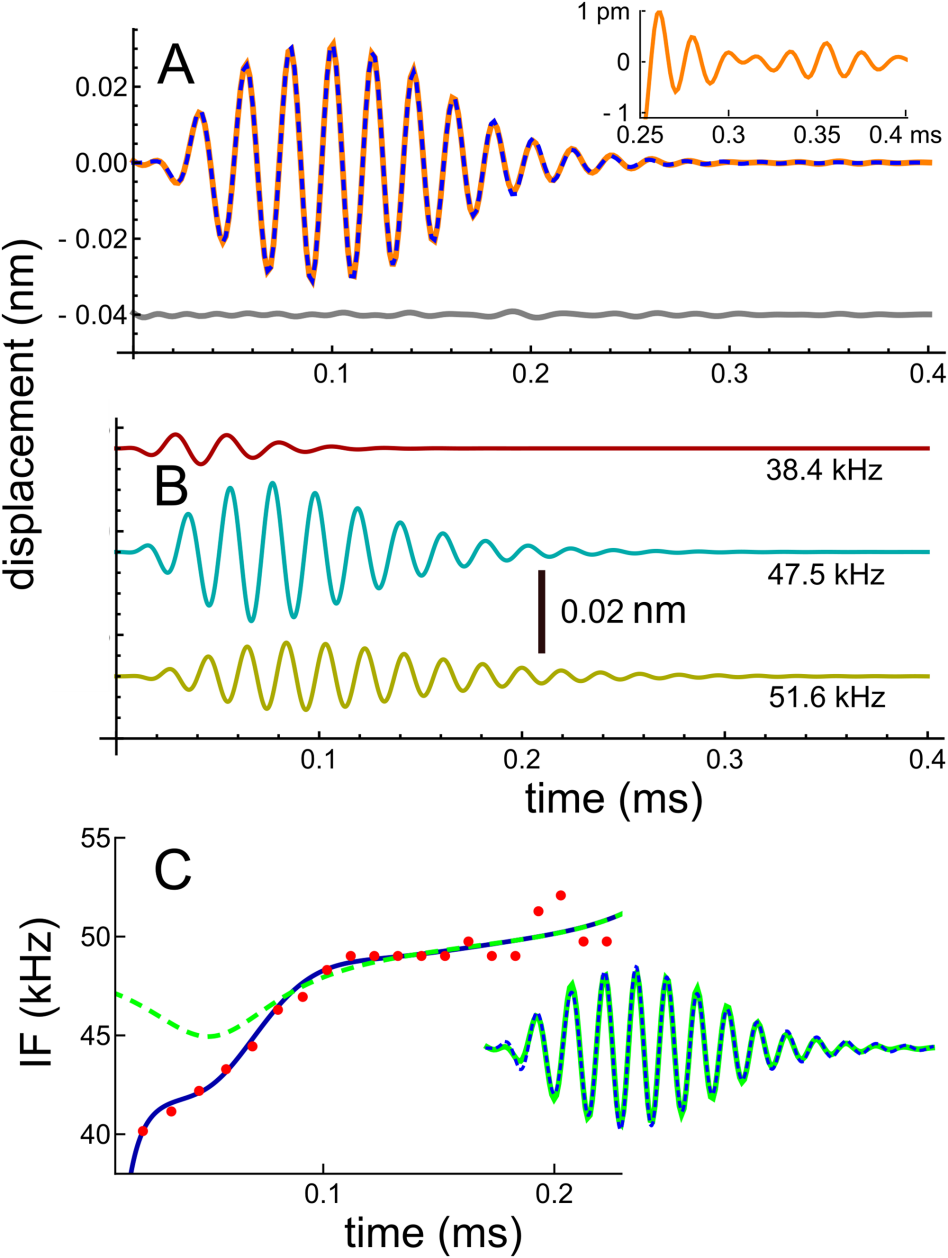
Impulse response of the basilar membrane of the mouse at a best frequency of 48 kHz (Ren, He & Barr-Gillespie, 2016), its three fitted gammatones, and the instantaneous frequencies. (A) Impulse response (orange line) fitted with the sum of three fourth-order gammatones of 51.6, 47.5, and 38.4 kHz (dashed blue line); the residual is shown in grey. Inset shows a magnified view of the later part of the waveform, where beating of multiple frequencies is evident. (B) The three gammatones shown separately. (C) The instantaneous frequency of the original waveform derived from zero crossings (dots), and the IF of the sum of the three gammatones (blue line). For comparison, the dashed line is the IF of the waveform derived from fitting just two gammatones (result in inset), indicating that all early components are important for accurately generating a glide.

In recent work, Ren, He & Barr-Gillespie (2016) studied the reticular lamina (RL) and BM of mice using a highly sensitive optical technique which involved shining a laser beam through the intact round window membrane. They measured displacement and phase responses to 30 dB SPL sinusoidal stimuli at a best frequency of 48 kHz. From the measured frequency domain responses, they obtained the corresponding impulse response (Fig. 2C of Ren, He & Barr-Gillespie (2016)) by inverse Fourier transform, and the data describing the waveform was kindly supplied by the primary author. The waveform is shown here in Fig. 2. The result of numerically fitting gammatones to the curve, as previously described, was that the waveform could be well represented as the sum of three fourth-order gammatones of frequencies 51.6, 47.5, and 38.4 kHz, also shown in Fig. 2, where the individual components are shown separately. In this case the frequency ratios between the three components are 1.09 and 1.24. In support of there being multiple frequencies within the waveform, we have magnified the tail of the impulse response and display it as an insert in Fig. 2A: it is clear that the waveform shows beating.

The IF of the actual waveform was calculated using zero crossings, and the upwards glide is shown in Fig. 2C as the red dots. For comparison, the IF of the combination of three gammatones was computed using the Hilbert transform, and the close match is shown by the continuous blue line. It can be seen that the early part of the waveform is responsible for the steepest part of the frequency sweep, and it is of interest that when the waveform was less accurately fitted—using just two gammatones—the largest error occurred at those early instants (see the inset in Fig. 2C, where the difference between the actual and fitted waveforms is just visible). The IF of the two-component waveform is shown by the dashed line, and in this case there is only a slight dip in the IF. This property sheds some light on the possible origin of the glide, an aspect addressed in the ‘Discussion’ (and Supplementary Material S1). This later analysis sets out why the IFs of the combination can show glides, even though the IFs of each of the component gammatones are constant over time. It is the specific combination of the gammatones—their beating—which in such cases leads to an observed glide. If the impulse response is sufficiently long-lasting, this same combination of gammatones also produces waxing and waning, as other cases below illustrate.

**Figure 3:**
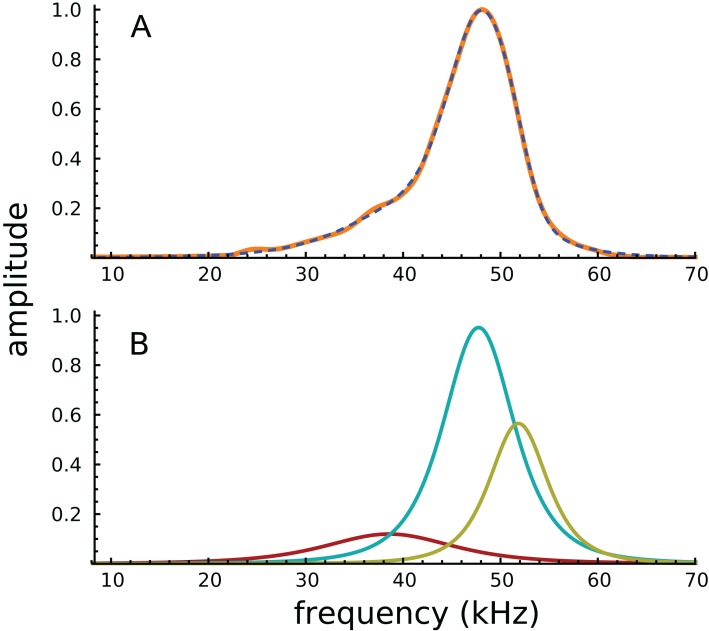
Amplitude spectra of the impulse response shown in Fig. 2. (A) The impulse response (orange) and the sum of the three gammatones (dashed line). (B) Colour-coded amplitude spectra of the individual gammatones.

Turning to the spectral domain, the spectra of the waveform and its component gammatones are shown in Fig. 3. The spectrum of the signal (orange line) is almost symmetrical about the best frequency of 48 kHz, with only a small amount of additional energy on the low-frequency slope, energy which appears to be the origin of the 38.4 kHz gammatone.

Because the frequency components are close together, and the impulse short, the Fourier transform shows only a single peak, with the other components effectively hidden. Of interest, all of Ren’s other acoustically evoked spectra, shown in the original publication, also display just a single peak with a slightly elevated low-frequency slope, as did spectra for the RL also recorded by Ren. When the RL data were subject to gammatone analysis, very similar results were obtained, with three gammatones of 36.9, 47.0, and 51.1 kHz appearing (the last two again giving a ratio of 1.09).

It appears that the SOG method, which relies on coordinated evolution of amplitudes and phases, is better able to resolve spectral components than the conventional Fourier transform. In support of this claim, it is noteworthy that some of the electrically evoked spectra shown in Ren, He & Barr-Gillespie (2016) do display double peaks (for example, there are peaks at about 46 and 52 kHz for a single animal in Ren’s Fig. 1B, a ratio of 1.13; and there is an average ratio of about 1.09 for five animals in Ren’s Fig. 1H).

The double peaks, which appear at a similar frequency ratio to the first two components separated by decomposition (1.09), support the suggestion that there are two actual frequency components acting on the BM, although they are not always able to be resolved in the frequency domain when the impulse response is short. However, the presence of two closely spaced frequencies, which when combined are apt to interfere, has implications for 2-DOF models and for the origin of glides. As outlined in the ‘Discussion’, the beating of two constant-frequency sine waves (or gammatones) can give rise to a series of frequency glides at times of destructive interference, and this mechanism is suggested as a possible origin of the glide seen here. Other mechanisms for the glide, including gammachirps (Irino & Patterson, 2001) have also been suggested, an option raised in a later ‘Discussion’ section on open questions.

### Case 2

**Figure 4:**
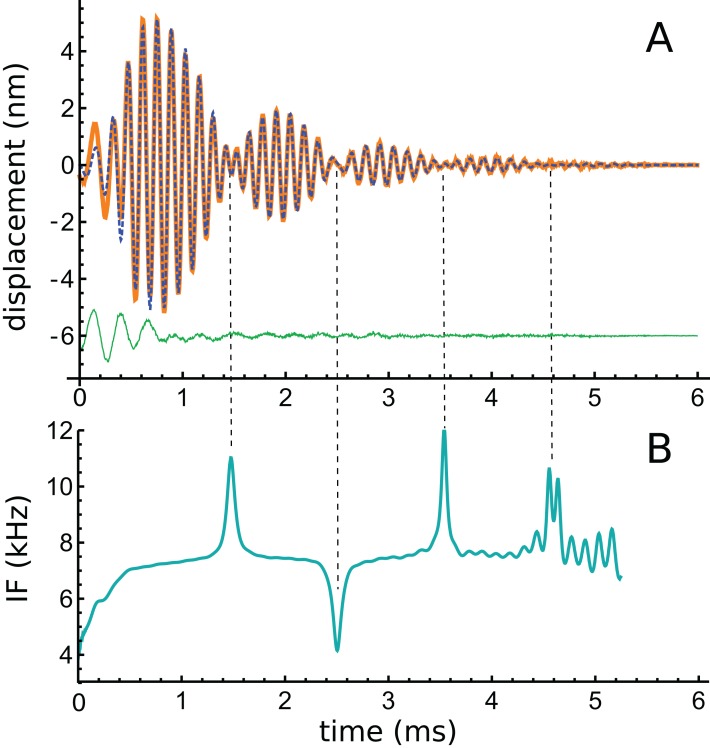
A multi-lobed basilar membrane impulse response from the chinchilla (Shera & Cooper, 2013) and its instantaneous frequencies. (A) Impulse response before (orange line) and after (blue dashed line) filtering. The green line beneath shows that the difference is mostly a short 4 kHz component. (B) Instantaneous frequency of the filtered signal as derived by Hilbert transform. There is an initial upward glide, followed by a prominent set of upward and downward sweeps at times when the waveform goes through amplitude minima (times marked with dashed vertical lines). Such upward and downward sweeps are characteristic of beating, as shown in Fig. A3 of Supplementary Material S1.

An impulse response comprising many lobes, and hence with the potential to test the limits of the 2-DOF model, is the waveform recorded by Shera and Cooper of the motion of a bead on the BM of a chinchilla (Shera & Cooper, 2013). The waveform (Fig. 4) shows distinctive waxing and waning, and has been the subject of three papers (Li & Grosh, 2016; Shera, 2015a; Wit & Bell, 2015), each with a somewhat different view of what it represents. It is of particular interest to the multiple-gammatone analysis because the lobe structure suggests it might be due to the beating together of constant-frequency gammatones. Consistent with this view, it was found that the phase of the waveform alternated between one lobe and the next, a characteristic of beating (Fig. A2 of Supplementary Material S1). Similar phase reversals between lobes have been previously noted in cochlear impulse responses (Shera (2001a), p. 1676 of Pfeiffer & Kim (1972)).

**Figure 5:**
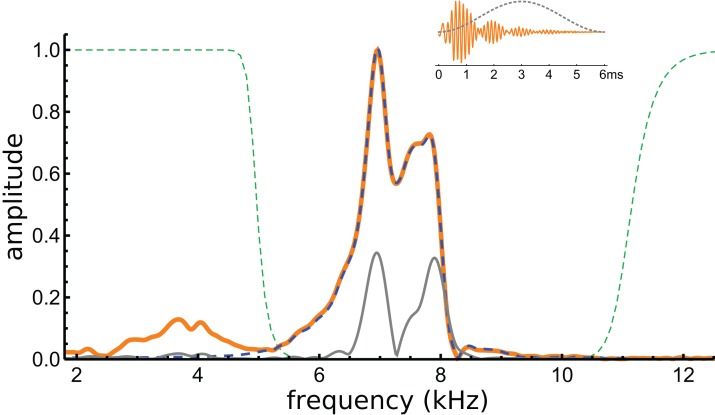
Normalised amplitude spectra of the Shera and Cooper signal before (orange) and after (dashed blue line) filtering. The dashed green lines are the profiles of the filters used to remove the low-frequency component and high-frequency noise. Note the notch at 8.3 kHz, which is also evident in Fig. 6C. The grey line is the spectrum of the signal after applying a Hann window (shown in the inset).

The original authors interpreted the waveform in terms of recirculating echoes, supporting the idea of multiple internal reflections (MIRs) and slow reverse traveling waves in the cochlea. A re-analysis by Wit & Bell (2015) pointed out that each of the five lobes in the waveform were not identical, implying that coherent reflection was perhaps not a complete description. In reply, Shera pointed out (Shera, 2015a) that the lobes themselves are not the repeating parts, instead the ‘atoms’ were long-lasting echoes, all starting at time zero, which extended across more than one lobe. When a suitable transfer function between one echo and the next is formulated, the sums of the successive echoes build up to give the original waveform (see Fig. 5 of Shera (2015a)), supporting coherent reflection theory. More recently, Li & Grosh (2016) considered the waveform to be made up of a ‘primary’ burst followed by a ‘secondary’ sequence (or ‘coda’) of individual wave packets, and their detailed finite element model was able to predict the core features of the waveform provided a suitable pattern of irregularity (a random degree of roughness) was introduced into the model. Without roughness, only the primary burst was reproduced, implicating this factor as a possible cause of the coda.

To explore the properties of the waveform in more detail, time–frequency analysis was performed. The data was first cleaned using lowpass and highpass filters (40th-order Butterworth) with cutoff frequencies of 5 and 11 kHz respectively, their effects being to remove a short 4 kHz component at the onset of the response and high-frequency noise. The waveform before and after filtering is shown in Fig. 4A, and the residual is also shown. Using the Hilbert transform, the envelope and IF of the waveform were calculated, and the result of the IF analysis is shown in Fig. 4B. The IF shows the usual initial upwards glide within the first 0.3 ms, but an obvious feature is a subsequent set of upwards and downwards sweeps at times (1.5, 2.5, 3.5, and 4.5 ms) when the waveform was passing through an amplitude minimum. The regular 1 ms gap between minima suggests that, after the first lobe, there appears to be beating of two frequencies differing by 1 kHz.

It is not widely appreciated that the beating of constant frequency components produces frequency glides at time of destructive interference. However, this property of beating was known by Helmholtz, and has been well described mathematically by Hartmann (1998), as set out in Supplementary Material S1. Figure A3 of S1 plots the IF of a beating pair of constant frequency sine waves and shows the typical upwards surge or downwards dip (depending on the relative amplitude of the components) at times of destructive interference. Together with the observed phase reversal between minima, the surges and dips in the IFs of the Shera and Cooper waveform suggest that the waxing and waning in the envelope might involve the beating of the fixed frequency components. On theoretical grounds presented by Lyon (2017), the components are likely to be gammatones, or at least gammatone-like. The relevance of these periodic surges in IF is provided in the ‘Discussion’ and Supplementary Material S2, where the impulse response of two coupled oscillators is shown to resemble a gammatone. The sweeps in IF are similar to those seen in other BM recordings (De Boer & Nuttall, 1997; Guinan & Cooper, 2008; Lin & Guinan, 2004) and suggest that the frequency glides studied by these authors might also involve the interference of two or more fixed frequencies.

If beating of gammatones underlies the waveform, it should be possible to identify the two component frequencies in its spectrum, and this formed the next stage of analysis. The spectrum of the waveform is shown in Fig. 5, and it can be seen that most of the signal lies in a band between 6 and 8 kHz. Three Fourier transforms were done, one on the entire signal, one on the filtered signal, and another on the entire signal multiplied with a Hann window (which emphasises behaviour in the middle of the signal and minimises early activity). Two large peaks emerge, one at 7.0 kHz and another at 7.9 kHz (see Fig. 5). The separation of about 1 kHz tallies with the minima at 1.48, 2.51, 3.54, and 4.60 ms, and is consistent with the beating model. To investigate more closely, the FindFit function in Mathematica was used to fit the sum of two gammatones to the later part of the waveform, and frequencies of 6.97 and 7.94 kHz emerged (a ratio of 1.14).

**Figure 6:**
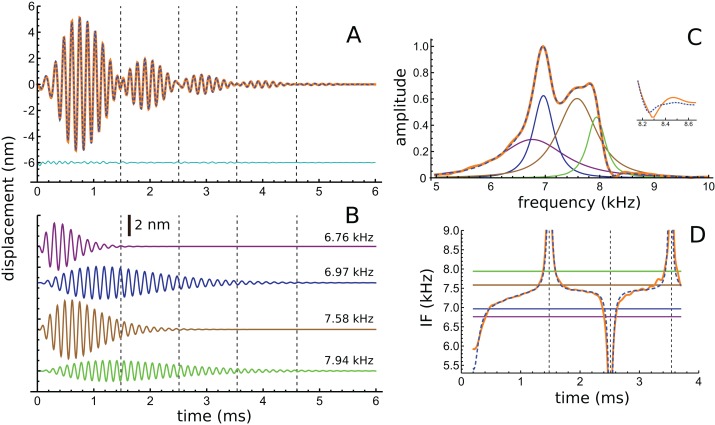
The impulse response of the Shera and Cooper signal fitted with the sum of four gammatones of order 3. (A) Sum of the gammatones (dashed blue line) compared to the original filtered signal (orange). The difference is shown underneath as the cyan line. Dashed vertical lines mark positions of envelope minima and align with peaks and dips in IF shown in Fig. 4B. (B) Profiles of the four gammatones of frequencies 6.76, 6.97, 7.58, and 7.94 kHz. Dashed vertical lines again mark envelope minima. (C) The spectra of the four colour-coded gammatones and of their sum (dashed blue line) compared to the spectrum of the filtered signal (orange). Note that the notch at 8.3 kHz is also reproduced (shown in detail in inset). (D) Instantaneous frequency of the sum of the four gammatones (dashed blue line) and IF of the filtered signal (orange) over the first 3.7 ms. The coloured horizontal lines show the invariant IFs of the individual gammatones. Parameters of gammatones: α_1_ = 141.2 ± 2.6, β_1_ = 5.76 ± 0.04, ζ_1_ = 6.671 ± 0.007, φ_1_ = −0.13 ± 0.02; α_2_ = 9.66 ± 0.17, β_2_ = 1.827 ± 0.008, ζ_2_ = 6.966 ± 0.001, φ_2_ = 4.74 ± 0.02; α_3_ = 62.5 ± 1.2, β_3_ = 3.45 ± 0.02, ζ_3_ = 7.583 ± 0.003, φ_3_ = 2.00 ± 0.02; α_4_ = 4.60 ± 0.08, β_4_ = 1.577 ± 0.008, ζ_4_ = 7.940 ± 0.001, φ_4_ = −1.14 ± 0.02. (Errors are derived from the least-squares fitting procedure of Abscissa).

In addition to these peaks, there are smaller peaks as well, and the fit routine was now used to fit the sum of four gammatones, with *n* = 3, to the filtered waveform. The outcome of the process is shown in Fig. 6, which shows how the filtered waveform can be accurately fitted with the sum of four gammatones of order 3, producing an rms error of 0.035 nm. Figure 6A shows the result of the fitting. Figure 6B shows the four gammatones, of frequencies 6.76, 6.97, 7.58, and 7.94 kHz, and illustrates their different amplitudes and decay rates. The respective frequency ratios are 1.03, 1.09, and 1.05. Figure 6C shows the spectra of the four gammatones individually and of their sum, and compares them with the spectrum of the filtered original waveform. It was found that gammatones of third order (*n* = 3) provided better fits than with *n* = 1, 2, or 4 (rms errors, respectively, of 0.062, 0.039, and 0.044 nm). The fitting procedure could be extended using more gammatones, but improvements were small. Together, four gammatones explain all the major features of the waveform and account for more than 97% of the total signal energy, the remainder being mainly in the 4 kHz component, which, for completeness, can be fitted with a fifth fast-decaying gammatone of 3.81 kHz.

Turning now to the IFs, consider first the times of envelope minima marked with the dashed vertical lines in Fig. 6A. The correspondence between these times and the occurrence of IF sweeps (Fig. 4) has already been pointed out. However, as shown by Hartmann (1998), the direction of the sweep when two waveforms destructively interfere depends on the relative magnitude of the components: upwards when the higher frequency has greater amplitude; downwards if it has lesser amplitude (Fig. A3). Applying this property to the later parts of the four gammatones isolated from the Shera and Cooper waveform explains the direction of the sweeps. Beyond 3 ms effectively only two gammatones—the one at 6.97 kHz (blue) and the other at 7.94 kHz (green)—contribute to the sum. Since the higher frequency gammatone has a slightly larger amplitude than the lower frequency one, it is to be expected (in accordance with Fig. A3) that the sweeps at 3.5 and 4.6 ms point upwards (Figs. 4B and 6D). At the earlier marks the situation is more complicated, because more than two gammatones are interfering. However, Fig. 6D shows that the IF of the sum of the gammatones (calculated by Hilbert transform) closely matches the actual IF of the filtered signal. In particular, the initial upwards frequency glide is well represented, even though the IFs of each of the four individual gammatones have constant frequency (horizontal coloured lines).

In summary, representing the impulse response as four component gammatones provides a way of explaining the total waveform’s time-domain, frequency-domain, and IF characteristics. Note that the spectral width of the gammatones is a reflection of their short time-spans, not any instability in their frequency. This supports the idea that the impulse response derives from four constant frequency components which beat together to produce waxing and waning; they also accurately reproduce the spectral profile and predict upwards and downwards glides in IF. Later discussion on the dynamical behaviour of coupled oscillators opens up the possibility that the set of gammatones may, directly or indirectly, be related to the way in which the masses of a coupled BM–TM system interact. Supplementary Material S2 shows that two coupled oscillators of identical natural frequency show waxing and waning when subject to an impulse, and they also exhibit glides; the ‘Discussion’ provides an historical overview of how waxing and waning, and glides, have been treated in the literature.

### Case 3

A range of impulse responses recorded in the chinchilla in response to clicks and tones were published in 2000 by Recio & Rhode (2000), data kindly made available by the first author. The responses came from microspheres placed on the BM near the round window and whose motion was measured with a laser interferometer. Many of the impulse responses showed waxing and waning, and all showed frequency glides. A valuable property of the data was that the responses were measured over a wide range of intensities and at various distances from the base.

**Figure 7:**
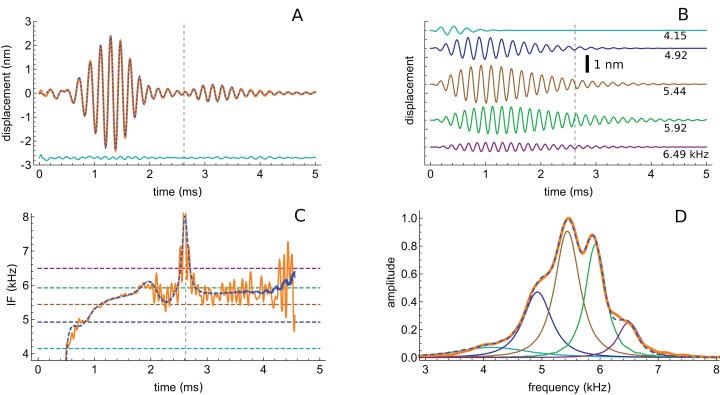
The impulse response from Recio & Rhode (2000) decomposed into five gammatones. (A) Impulse response to a 56 dB SPL click (orange) and the sum of five third-order gammatones (dashed blue line). The dashed vertical line marks the position of the minimum in the signal envelope at 2.6 ms where destructive interference of the gammatones occurs. (B) The separate gammatones with frequencies of 4.15, 4.92, 5.44, 5.92, and 6.49 kHz. (C) Instantaneous frequency of the signal (orange) compared to the IF of the sum of the gammatones (dashed). (D) Spectrum of each of the gammatones (colour-matched lines) and of their sum (dashed) which gives a good match to the spectrum of the total signal (orange). Data from Fig. 3 of Recio & Rhode (2000).

The first impulse response to be analysed originated from a position with a CF of 5.5 kHz (their Fig. 3). The waveform in response to a 56 dB click is shown in Fig. 7A, and a similar fitting sequence to before was employed. After first fitting the tail of the response with two gammatones of about 5.4 and 5.9 kHz, it was found that the total signal could be accurately decomposed into five third-order gammatones (4.15, 4.92, 5.44, 5.92, and 6.49 kHz; ratios of 1.19, 1.11, 1.09, and 1.10 respectively), which are shown separately in Fig. 7B.

The total spectrum (orange line in Fig. 7D), closely approximates the spectrum of the sum of the individual gammatones (dashed blue line). In this case there is a good fit, both temporally and spectrally, using five gammatones. Once again it is worth noting that the width of the gammatone spectra reflects only their short time-span, not any underlying frequency instability. Since gammatones have constant frequency over time (dashed horizontal lines in Fig. 7C), this implies that beating could take place over the full duration of the impulse response, with the recorded waveforms deriving from the combined vibration of multiple stable oscillators in the cochlea. In support of this possibility, Fig. 7A shows that there is an envelope minimum at 2.6 ms, and Fig. 7C shows that there is a spike in IF at this exact time, corresponding to destructive interference (Fig. A3 of Supplementary Material S2). Figure 7C also shows that the IF of the sum of the gammatones (calculated by Hilbert transform, dashed blue line) accurately tracks the IF of the original signal (orange line), not just at 2.6 ms but over most of its course, including the initial glide.

The success with which gammatones can be fitted to the waveform suggests that the signal contains multiple components with coordinated amplitudes, frequencies, and phases, features that cannot be revealed by standard Fourier analysis. The successful decomposition supports the idea that the gammatones could, at least approximately, reflect the activity of some sort of coupled oscillating system that might usefully form the basis of a more accurate 2-DOF lumped-element model (Ni et al., 2014).

**Figure 8:**
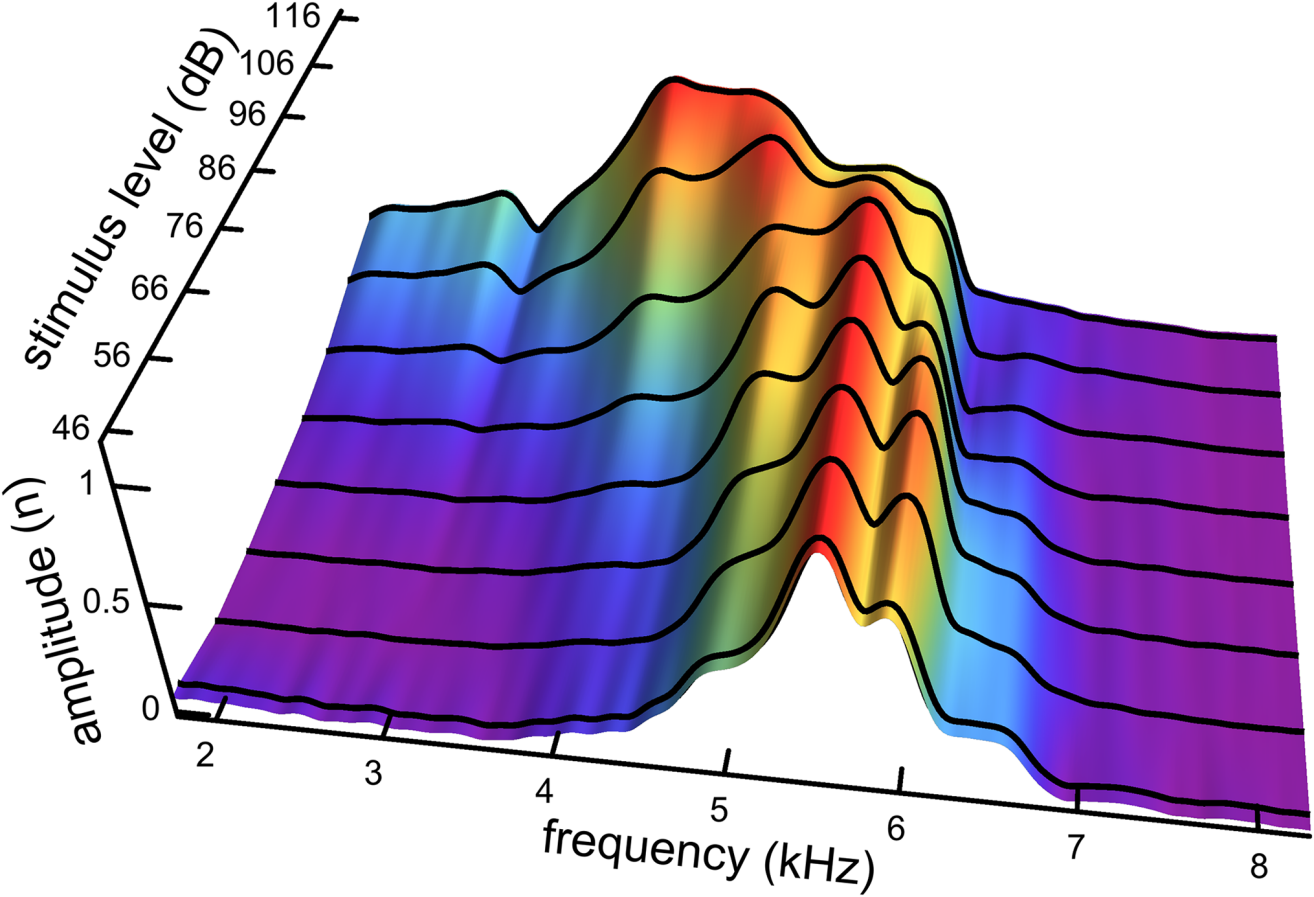
Normalised spectra of the impulse responses (black lines) as click intensity rises from 46 to 116 dB SPL. The set of parallel ridges indicates the presence of fixed underlying frequencies. There are five or six spectral peaks, and so the waveform at a given click level can be well fitted with five or six gammatones carrying the frequencies of the peaks (as in Fig. 7). Data from Fig. 3 of Recio & Rhode (2000).

**Figure 9:**
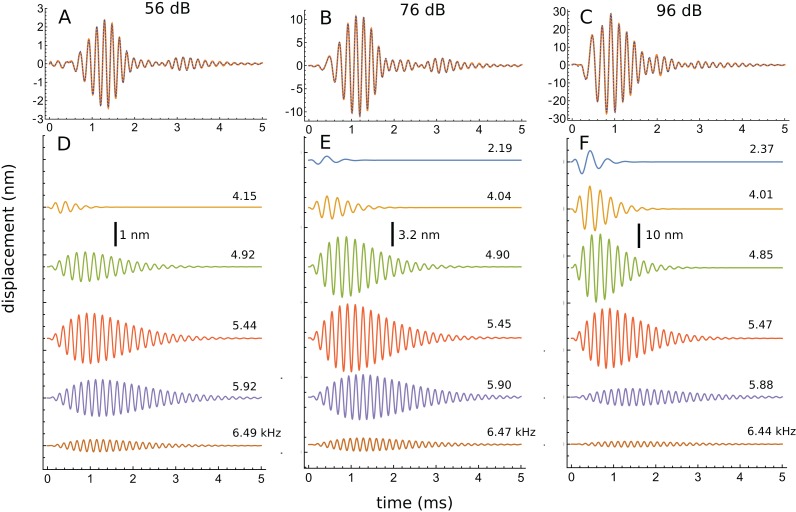
Frequency stability of the fitted gammatones as intensity of the impulse responses increases. (A–C) Impulse responses (orange) recorded at 56 dB, 76 dB, and 96 dB and the sum of the fitted gammatones (dashed). (D–F) The individual third-order gammatones. At 56 dB (D), five gammatones are sufficient, but an additional gammatone at about 2.2 kHz emerges at 76 and 96 dB (E and F). Note that all the gammatones remain fixed in frequency, as the labels indicate. Note also that the gammatones are shorter (low *Q*) at low frequency and longer (high *Q*) at high frequency. In addition, as intensity rises, low-frequency gammatones become progressively larger (in relative terms) and higher frequency ones progressively smaller. Data from Fig. 3 of Recio & Rhode (2000).

An interesting aspect of the spectrum in Fig. 7D is that the 4.92 kHz component is almost hidden within the low-frequency slope (a reflection of its closeness in frequency and its short time-span, a property seen in other impulse responses examined and the likely reason that such components have escaped notice). Also partly hidden is a small and broad contribution at about 4 kHz, which the SOG method interprets as an additional short-lived and weak source at this frequency. It is therefore of interest to observe what happens when the click intensity is increased. Figure 8 is a normalised 3D plot of all the data from 46 to 116 dB and it shows that at higher intensities (76–116 dB), a clear additional gammatone component emerges at 4.1 kHz. Importantly, as the additional peak emerges, the other pre-existing peaks stay at their previously determined frequencies, as shown by the parallel ridges in Fig. 8. Figure 9 also casts light on the matter, indicating the exceptional frequency stability of the gammatones as intensity increases. This plot analyses the impulse responses at 56, 76, and 96 dB into their constituent gammatones, and the frequency labels in Figs. 9D–9F show that the frequencies change by as little as 1%. This is evidence supporting the view that the gammatones are not artefacts of the fitting procedure.

Another perspective gained from Fig. 9 is that, as intensity grows, the peaks below CF gradually increase in relative terms, while those above CF steadily diminish. Since the low-frequency components are short (low *Q*), while the high-frequency components are much longer (high *Q*), this means that the overall *Q* of the cochlea tends to decrease as intensity rises—the impulse responses appear shorter, and the weighted peak of the total response shifts to lower frequencies, consistent with what is experimentally observed.

A similar decomposition technique was applied to other Recio and Rhode data (their Fig. 2, with CF of 14.5 kHz), and a generally similar pattern emerged. There were consistent spectral peaks, good fits to the sums of 5 or 6 gammatones, similar ratios between frequencies, and, as intensity rose, a relative increase of low frequency peaks and dwindling of high frequency peaks. However, the data was considerably noisier and there were multiple small peaks between the major ones.

### Case 4

**Figure 10:**
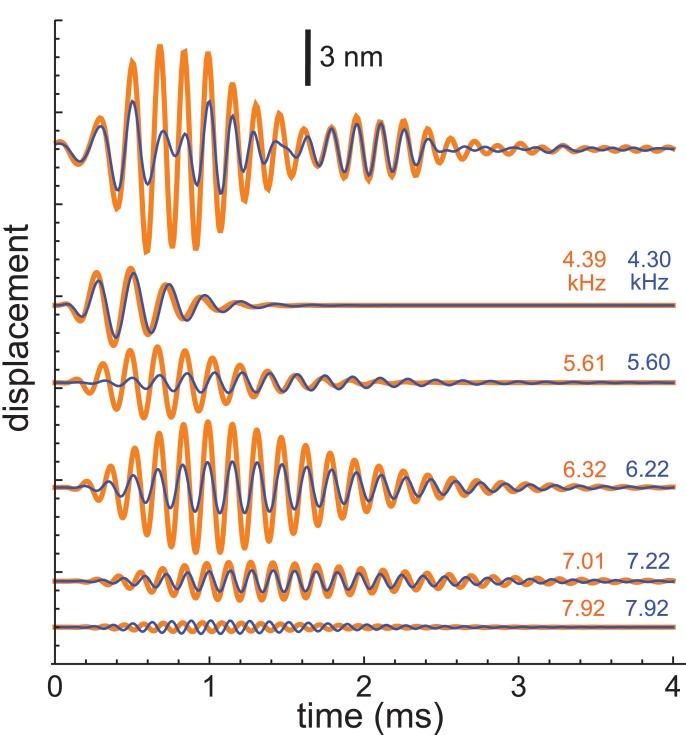
Impulse responses from the chinchilla, obtained without suppression (orange) and in the presence of 30 dB of Gaussian noise (blue). At top are the actual waveforms, and below are the colour-coded component gammatones, with labels indicating their frequencies. Data from Recio-Spinoso & Cooper (2013).

More recently, Recio-Spinoso and Cooper used a laser interferometer to record impulse responses from the chinchilla and gerbil (Recio-Spinoso & Cooper, 2013), and the first author kindly made the data available for analysis. This work involved recording two types of impulse responses, one obtained in response to a click only (that is, with a quiet background) and another obtained in response to a similar click but with added background noise. Examples of the two sorts of responses (for a chinchilla with CF of 6.8 kHz) are shown at the top of Fig. 10, and it is evident that the waveforms differ considerably, with the added Gaussian noise producing a clear reduction in amplitude (a suppression effect which was the main focus of the Recio-Spinoso and Cooper study).

For the present work, the interest was in seeing whether the SOG approach could be consistently applied to both conditions and whether differences in the traces could be attributed to particular features in the extracted gammatones. The impulse responses both came from the same bead at the same location on the BM of the same animal, so the mechanical basis of the waveforms should be identical. Both waveforms, which showed clear evidence of waxing and waning, were subject to gammatone analysis as before.

Each waveform could be accurately analysed into the sum of five fourth-order gammatones, and each of these components is also shown in Fig. 10 (orange for the unsuppressed case; blue for suppressed). The notable finding was that, despite the different form of each impulse response, the frequencies of all the component gammatones were nearly the same, as the labels on the traces indicate. Only the amplitudes of the component gammatones differed appreciably, with suppression mainly affecting the 6.3 and 5.6 kHz components. The result was that suppression was largely limited to the first lobe of the impulse response, with the second lobe remaining nearly the same. The end result was similar to what was found before, where the first lobe was largely comprised of short-lived, low-frequency gammatones, and the second lobe was made up of long-lasting, high-frequency gammatones. Zero-crossings of both the compound waveforms, as well as all the individual components, stayed relatively fixed between the unsuppressed and suppressed cases. This applies even to the two 7.92 kHz components, which are out of phase.

**Figure 11:**
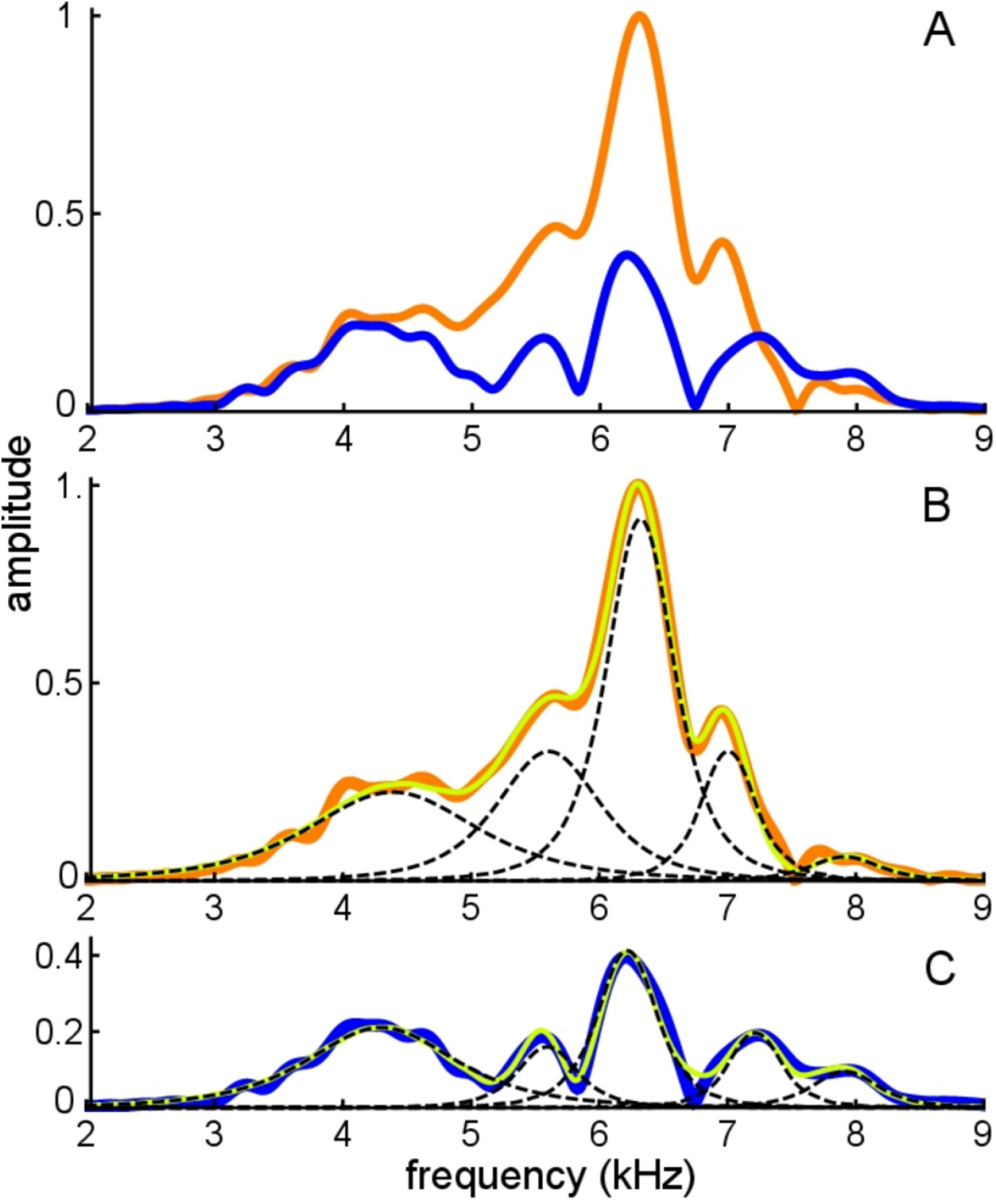
Frequency domain view of Fig. 10. (A) Amplitude spectra of the experimentally recorded waveforms: unsuppressed impulse response (orange) and suppressed impulse response (blue). Five main spectral peaks are evident. (B) Match between the amplitude spectrum of the sum of the fitted gammatones (yellow) and the original spectrum (orange) for the unsuppressed case. The amplitude spectra of the five individual gammatones are shown as dashed black lines. (C) Suppressed case, with the original spectrum in blue, amplitude spectrum of the sum of the fitted gammatones in yellow, and of component gammatones in black.

The average frequencies shown in Fig. 11 are 7.92, 7.12, 6.27, 5.61, and 4.35 kHz, giving ratios between intervening gammatones of 1.11, 1.13, 1.12, and 1.29, values in line with those found before.

Figure 11A shows both the unsuppressed and suppressed cases in the spectral domain, and here it is evident that there are five dominant peaks which appear at nearly the same frequencies in each case. Figure 11B and 11C shows that each peak corresponds to a component gammatone. Although the frequencies of these gammatones stay virtually fixed, their amplitudes change markedly between the suppressed and unsuppressed cases, except for the lowest component at 4.3 kHz which is almost unchanged. As with previous findings, when all the gammatones are added together, the spectrum of the sum gives a good fit to both the suppressed and unsuppressed profiles—notably the peaks and troughs—showing the usefulness of the SOG approach.

### Case 5

The analysis here examines the impulse response of the cochlear model constructed by Elliott, Ni & Sun (2017). The model is comprised of multiple fluid-coupled sections each of which has 2-DOF micromechanics involving the BM and the TM. The model’s response was designed to fit the noninvasive optical coherence tomography data obtained by Lee et al. (2016) for the mouse cochlea, and the findings were that a model based on 2-DOF mechanics fitted the data better than a single degree-of-freedom model. The impulse response of the model was calculated by Elliott et al. (Fig. 7 of their paper) for various levels of excitation and for 1D or 3D fluid coupling. The data for the simpler 1D case was kindly provided by the authors, and the question of interest was how well the impulse response could be fitted with gammatones.

**Figure 12:**
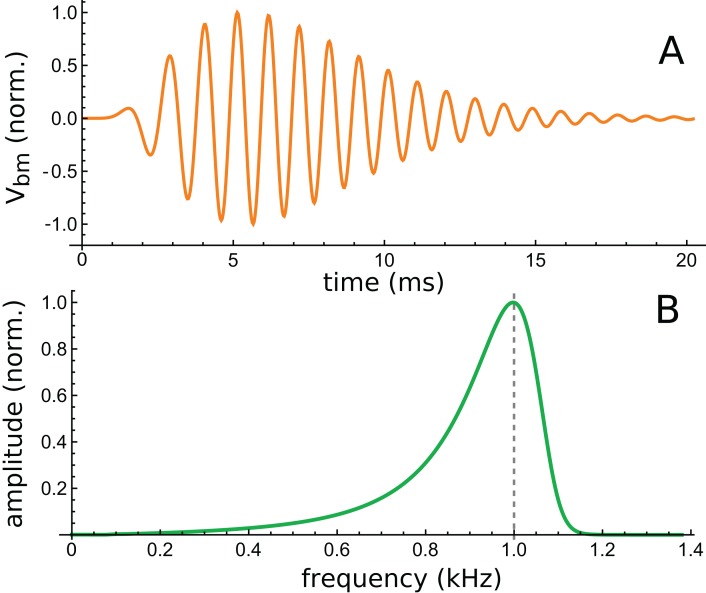
Time and frequency domain views of the impulse response of a 2-DOF cochlear model with 1-D fluid coupling (Fig. 7 of Elliott, Ni & Sun, 2017). (A) The impulse response for CF of 1 kHz and 10 dB excitation (data courtesy of the authors). (B) Calculated amplitude spectrum of this response.

The lowest intensity (10 dB) curve was chosen for study because its response was the longest lasting. This curve is shown in Fig. 12A, together with its calculated spectrum (Fig. 12B).

**Figure 13:**
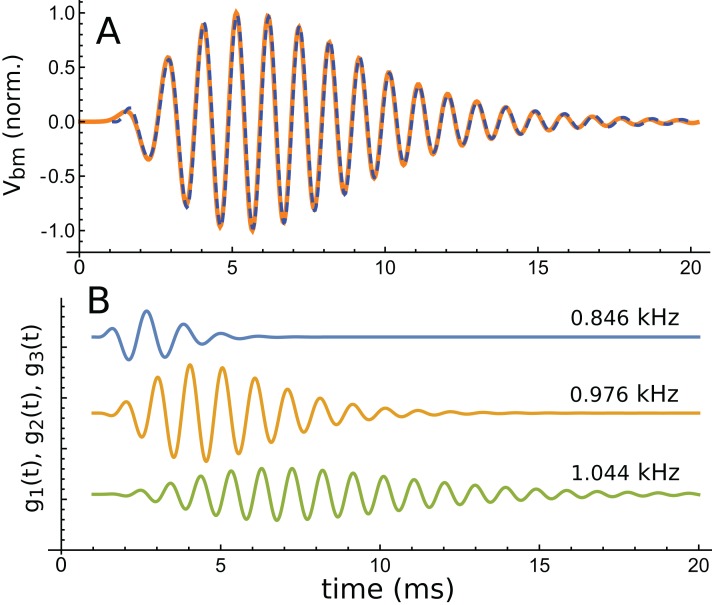
The same impulse response of Fig. 12 and its fitted component gammatones. (A) The impulse response (orange line) is well fitted with the sum of three gammatones of order 4 (dashed blue line). A time offset of 1 ms was selected by eye, and later fits done with the delay parameter left free confirmed this was appropriate. (B) The three gammatones shown separately. Their frequencies are 1.044, 0.976, and 0.846 kHz, giving neighbouring ratios of 1.07 and 1.15. Fitting parameters: α1 → 2.894, β1 → 1.958, ζ1 → 0.8462, φ1 →–2.667, α2 → 0.5422, β2 → 0.9124, ζ2 → 0.9761, φ2 → 0.2326, α3 → 0.0544, β3 → 0.5223, ζ3 → 1.0439, φ3 → 3.036. Using gammatones of order 3 or 5 made little difference to the fitted frequencies.

Using the fitting procedure described in the ‘Methods’, the chosen waveform was fitted with a series of three gammatones of order 3, that is, g(*t*) = α*t*^2^ exp(−β*t*) cos(2πζ*t* + φ). The result is shown in Fig. 13A, where a comparison is made between the impulse waveform (orange) and the sum of the three gammatones (dashed blue line). The three gammatones are shown separately in Fig. 13B. Their frequencies are 1.044, 0.976, and 0.846 kHz. The ratios between these frequencies are, respectively, 1.07 and 1.15. The outcome is very similar to what was seen in Case 1, since both derive from non-invasive measurement of the mouse cochlea.

A fit to the 30 dB waveform was also done, and it again showed that three gammatones provided an accurate fit. The three gammatones had frequencies of 1.049, 0.964, and 0.769 kHz. This represents next-neighbour ratios of 1.09 and 1.25, respectively. The other higher-intensity impulse responses (at 50 and 70 dB) were short, and fits were not attempted.

**Figure 14:**
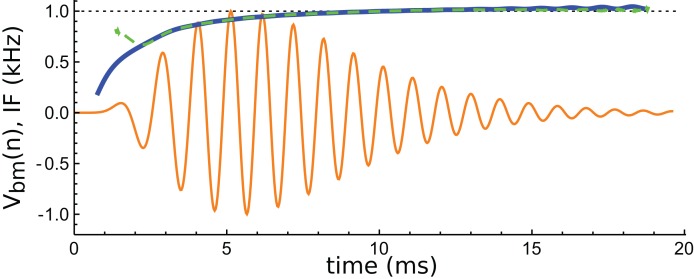
Instantaneous frequency (IF) of the impulse response shown in Fig. 13 calculated by Hilbert transform. The continuous blue line is the IF of the original waveform, and the green dashed line is the IF of the sum of the three component gammatones. The IFs are well matched, except at the very beginning.

An interesting aspect of cochlear impulse responses is that they show initial glides—an upwards sweep in frequency at the beginning of the waveform. There have been a number of explanations offered for glides (Shera, 2001b), and a fuller treatment is provided in the ‘Discussion’. Here it is noted that the impulse responses presented by Elliott et al. all showed glides, and an example is shown in Fig. 14. Note the typical upwards sweep in frequency over the first few cycles (blue line in Fig. 14).

A feature of the glide is that it is replicated in the synthetic waveform—that is, when the Hilbert transform is applied to the sum of the three gammatones, a similar glide emerges (green dashed line in Fig. 14). Since each individual gammatone has constant IF the glide in the combination comes from the beating of the individual components. Supplementary Material S2 confirms how the beating of constant frequency signals (even sine waves) leads to glides at instants of destructive interference. As mentioned in the ‘Discussion’, some authors have taken glides as evidence of beating in the cochlea, although others have treated them as an indication of some dispersive process. The glide examined here, and those in the previous examples, support the beating hypothesis.

The lingering question is what physical component within Elliott’s 2-DOF model might produce a gammatone, and this is the focus of the next section.

### Case 6: Two coupled oscillators—relationship to gammatones

**Figure 15:**
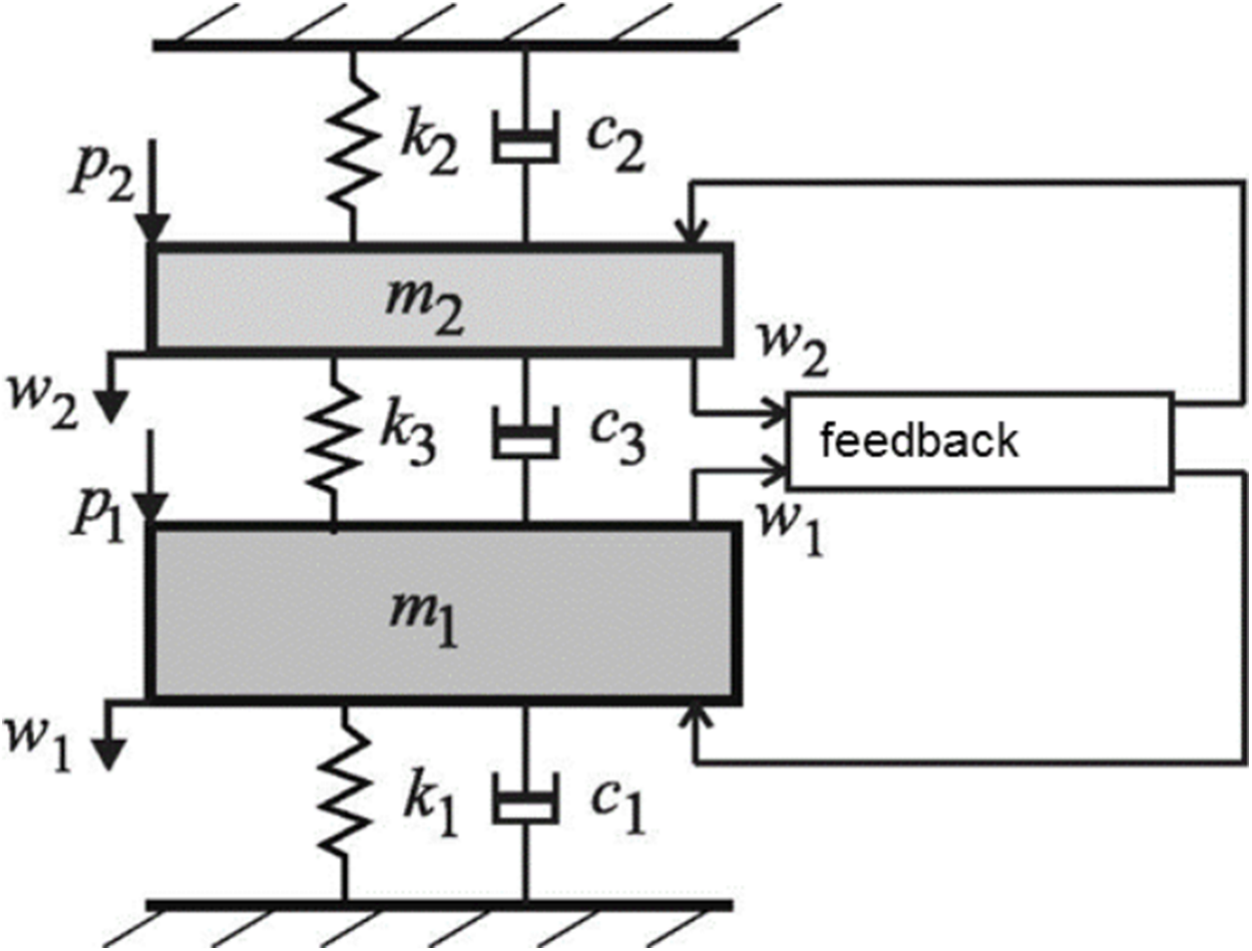
An active 2-DOF micromechanical model of the cochlea as an elastically coupled two-mass system. One mass, *m*_1_, is taken to be the basilar membrane; the other mass, *m*_2_, is taken to be the tectorial membrane. Between them is an active feedback loop. Image credit: modified from Elliott, Ni & Sun (2017), with permission.

Physically, the basic model for a 2-DOF system is two coupled oscillators, and the general form of the model is shown in Fig. 15. The system comprises two coupled masses, *m*_1_ and *m*_2_, where the first is identified with the mass of the BM and the second the mass of the TM. Not only are the two masses coupled by a compliance, there are also feedback forces involved, which Elliott, Ni & Sun (2017) formulated in terms of an active feedback gain parameter. These workers found that the 2-DOF model gave a better fit to actual cochlear data (as measured noninvasively with optical coherence tomography of the mouse cochlea by Lee et al. (2015)) than a simpler single degree-of-freedom model, which uses just a single local mass.

Elliott and co-workers framed their model in terms of its poles and zeros, a strategy first developed by Zweig (1991) and subsequently elaborated (Zweig, 2015, 2016). A noteworthy aspect of the work by Elliott et al. is that their 2-DOF model can be reduced to two pairs of closely spaced poles and one pair of zeros, a total of just seven parameters in all (an overall mass, and the frequencies and damping of the three pole/zero combinations). They highlight the important result that the two pairs of poles are almost coincident (p. 672), with the implication that the undamped natural frequencies of the admittance poles are nearly equal—which might be interpreted to mean that these two frequencies could combine and produce beating.

In this section the impulse response of the basic 2-DOF arrangement (Fig. 15) is calculated, and it turns out that the response of the second oscillator can be well represented by a combination of two second-order gammatones. The IF of the second oscillator is also found to go through a series of IF sweeps very similar to the glides observed in the cochlea. The model of two coupled oscillators is therefore put forward as an explanation for why the SOG approach appears to work: two coupled masses are the simplest form of a 2-DOF model, and the impulse response of the second mass (the BM) resembles a gammatone.

**Figure 16:**
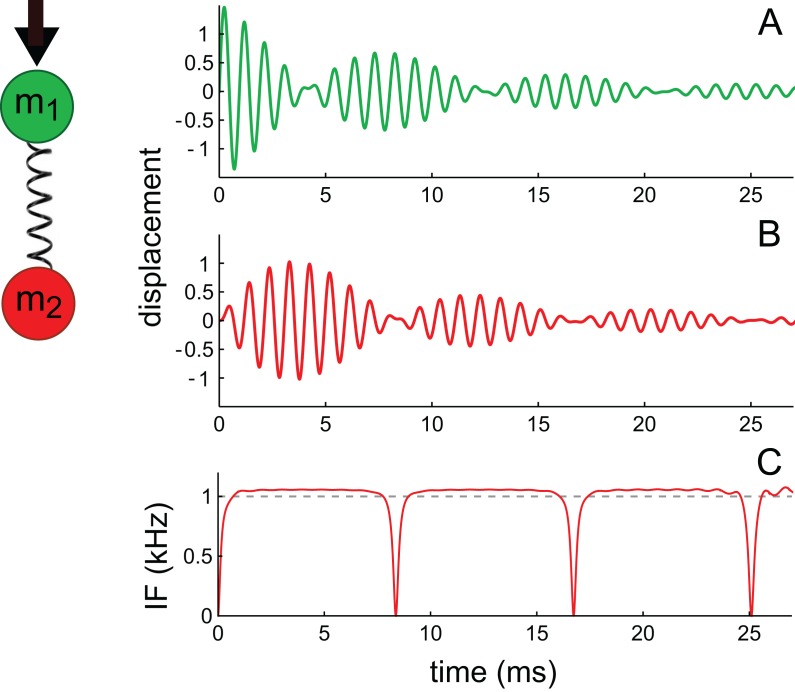
Two elastically coupled masses, *m*_1_ and *m*_2_, and their displacements (A), (B) in reaction to an impulsive force applied to *m*_1_. Mass *m*_1_ is associated with the tectorial membrane (experimentally unobserved), and *m*_2_ with the basilar membrane (observed); both have identical natural frequencies of 1 kHz. The spring includes a damping parameter. Note the beating-like waveforms in which each oscillator exchanges energy with its companion (the total energy is shared between them). The displacement of *m*_2_ (shown in B) is similar to impulse responses of the basilar membrane (including phase alternation between lobes), and can be approximated with the sum of two second-order gammatones. The IF of the waveform in (B) (shown in C) is similar to cochlear glides (e.g. as seen in Fig. 4B) and to the IF of the beating waveform shown in Fig. A3 of Supplementary Material S1. The waveforms reflect the equations derived in Supplementary Material S2 using the parameters γ = 0.1, ω_0_ = 2*π*, and κ = 5.

The first oscillator, identified as the primary oscillator, receives the initial impulse, and the other oscillator, elastically linked to it, is the secondary oscillator. Following suggestions summarised in Richardson, Lukashkin & Russell (2008), the first oscillator is taken to be a mass–spring system arising from the TM; the second oscillator is identified with the BM and it undergoes forced oscillation via its coupling to the first. The TM–BM pair therefore exchange energy as in a coupled pendulum. The simultaneous equations to be solved are given in Supplementary Material S2, and the solutions are displayed in Fig. 16. (Incidentally, because of symmetry, it doesn’t matter if *m*_1_ and *m*_2_ are interchanged, as was done by Elliott et al.).

As shown in Fig. 16, the coupled oscillator pair exhibits three distinctive properties.

First, the pair trade energy back and forth, their amplitudes waxing and waning in a similar way to two beating sinusoids. The first oscillator in the pair, excited with a velocity impulse, begins with maximal displacement during the first cycle and subsequently decays; the second oscillator, in contrast, begins with zero displacement and then, driven by the first, increases its oscillation before it too decays. The pair exchange energy until it is all dissipated in resistive losses. This pattern is broadly similar to those seen in the previous five cases. Beats originate from interference between the normal modes of the system, so even if the two oscillators have identical masses and natural frequencies, beating will occur (Ingard, 1988). The difference frequency (beat frequency) between the normal modes relates directly to the strength of the elastic coupling between the masses—the stronger the coupling, the higher the beat frequency.

A second characteristic feature is that the IF of the second mass goes through a pattern of glides (Fig. 16C) which is again similar to that found in the previous case studies. The analysis shows that the glides come about from beating of the gammatone components—in the same way as happens with the beating of sines (Supplementary Material S1).

Thirdly, the envelope of the second oscillator resembles a multi-lobed cochlear impulse response, and the waveform of the displacement can be approximated by a combination of gammatones, as theory and numerical curve-fitting indicates (Supplementary Material S2). Theoretically, the exact solutions are actually first-order gammatones—decaying sines—but the shape of the response of *m*_2_ can be approximated with the sum of two second-order gammatones.

## Discussion

The inner workings of the biological spectrum analyser within the ear are still a matter of debate, but impulse responses provide important clues. Indeed, a key test of any cochlear model is how well its impulse response mimics that of the actual cochlea, and here we have found that, using an SOG approach, cochlear impulse response can be well replicated by the sum of a set of two to six closely spaced gammatones. But what is the significance of this finding?

The connection between impulse responses and gammatones is suggested by filter theory (Lyon, 2017) and closely spaced poles appear in 2-DOF models of the cochlear partition’s cross-section (Elliott, Ni & Sun, 2017). Yet the cochlea is a distributed system, not a collection of lumped elements (Lyon, 2017, Ch. 12), so finding signs of discrete elements—gammatones—calls for further investigation. Perhaps some form of lumped-parameter model, suitably elaborated, may be appropriate (Ni et al. (2014)). Any continuous (distributed) system can be transformed into a discrete model using numerical methods, and these approaches may be useful. Recent research has found that the cochlea has a spiral ‘staircase’ structure in which there are fixed steps separated by a frequency ratio of about 1.06 (Shera, 2015b). The gammatones found here are separated by a similar ratio, so it is possible that, speculatively, each of the staircase’s quantised steps contributes in some way to the impulse response. Other work has also pointed to quantised cochlear behaviour; for example, Wit & Van Dijk (2012) found that coupled cochlear oscillators tended to cluster together to form frequency plateaus, with the steps having a comparable ratio.

A core question is why some impulse responses require only two or three gammatones whereas others need as many as six. Coupling of the BM and TM accounts for two gammatones, but more than this is hard to explain. In all the examined cases the prevailing frequency ratio was about 1.1, a finding that implicates the staircase structure of the cochlea, but which also suggests that the underlying mechanics is not local but extended. In such a situation, one might turn to traveling wave dynamics (Shera, 2015b; Zweig, 1991, 2015), electrical coupling (Nankali et al., 2018; Zweig, 2016), or consider that the resonances are directly excited by fast-acting sound pressure (Bell, 2012, 2014; Bell & Wit, 2015). In Bell & Fletcher (2004) the suggestion is made that resonances could occur as standing waves between rows of outer hair cells. Although traveling waves provide dispersion, and in turn readily explain glides, the difficulty is that all the gammatones used here begin at time zero, implying that all the resonances have nearly instantaneous physical connections—inconsistent with the progressive delays of a traveling wave. Further consideration of these difficulties is addressed in the last section below.

### Real or artefacts?

A possible criticism is that all the gammatones we have found are not real but simply artefacts of the fitting process. Against that view, the following observations can be presented. The extracted gammatones show consistently small ratios (1.05–1.15), the same ratio as often noted in the cochlear literature (Bell & Jedrzejczak, 2017; Shera, 2015b; Wit & Van Dijk, 2012). This small ratio explains why the observed number of waves within the later lobes tends to be about 10, as dictated by the beating equation of Eq. (A6).

Moreover, the gammatones are found to be stable entities, in that the extracted frequencies do not change when the intensity of the click is varied (46–116 dB, Case 3) or when suppressive white noise is added (Case 4). In these examples, the envelopes of the impulse responses changed appreciably, but the component gammatone frequencies nevertheless stayed fixed. Moreover, the very same gammatones which explain waxing and waning in terms of beating are also able, without any change in parameters, to explain the initial glide and the complete IF profile. It is notable that the spectral notch seen in the impulse response of Case 2 can be simply explained as the sum of the underlying gammatones.

Nevertheless, even if the first few isolated gammatones do correspond to actual resonances, the SOG method is unable to say whether further isolated gammatones are physical resonances or artefacts due to overfitting. This is a major limitation of the present work, and without further experimental exploration the boundary between what is real and what is unreal must remain indistinct. We leave it to further study to resolve the status of the resonances identified by the SOG procedure.

However, for the reasons given above, it appears that at least the first few gammatones relate to actual resonances, and the following text examines some implications. In particular, there are two cochlear impulse response properties—waxing and waning, and glides—which have been treated as separate phenomena in the literature. The following two sections focus on how the SOG approach can explain these two distinctive features in terms of just the one phenomenon, beating.

### Waxing and waning

Waxing and waning has been observed under a number of different conditions and has been remarked upon by a number of workers (Recio et al., 1998; Lin & Guinan, 2000; Robles & Ruggero, 2001; Shera, 2001b; Lin & Guinan, 2004; Guinan & Cooper, 2008). Initially, the present work began by considering the impulse response published by Shera and Cooper (Fig. 9 of Shera & Cooper (2013); Case 2), which resembles a beating waveform. This led to a re-examination of the iterated echo or MIR model (Wit & Bell, 2015), and in turn a rejoinder by the original lead author (Shera, 2015a). Usefully, the rejoinder explicitly displays the waveforms of each of the putative recirculating echoes and the relationship between them: it makes clear that each echo is not an individual lobe but rather a distributed waveform extending across multiple lobes. This section compares and contrasts this interpretation with that of the beating model.

The MIR interpretation Shera (2015a) is built on a detailed and complex mathematical framework which, in our view, is still not able to fully explain the fine spectral features of the impulse response or its accompanying glide. In particular, as set out in the text of Case 2, the MIR approach tends to minimise the significance of multiple spectral peaks, the deep spectral notch at 8.3 kHz, and the recurring pattern of frequency glides (Fig. 4B)—features supporting the beating model.

To explain the spectra analysed here as Case 2, Shera’s approach is to use a smooth transfer function which combines the two distinct peaks at 7.0 and 7.9 kHz into a single broad peak at about 7.4 kHz (see Fig. 1B of Shera (2015a)). In contrast, the beating model takes these two individual frequencies to be important, and considers them as the origin of a 1 kHz beating frequency. Fitting gammatones to Case 2 shows that the two peaks represent discrete gammatones of 6.97 and 7.94 kHz (Figs. 5 and 6B) and the 1 kHz difference frequency produces the 1 ms waxing and waning cycle.

The second aspect that the MIR model cannot explain is the presence of the deep spectral notch at 8.3 kHz (Fig. 1B of Shera (2015a)). Shera finds the notch troublesome, for it produces a corresponding large spike in the transfer function, |*H*|, which is sometimes greater than unity (Fig. 3 of Shera (2015a)). The author says the notch has little functional consequence, and applies a 40th-order low-pass filter at 8.2 kHz in order to remove it. However, there is no need to eliminate the notch, as it can be interpreted in terms of the beating model. The notch frequency is precisely predicted by the beating model: analysis of the original data shows it is formed by the destructive interference of multiple gammatones whose individual presence is evident as distinct spectral peaks (Figs 5 and 6C).

The beating model was first put forward by Lin & Guinan (2004). These authors describe how beating involves multiple component frequencies (their Fig. 1), whereas echoes in an MIR model involve wave bursts of a single frequency but of generally different phases (their Fig. 2). Their analysis of BM and auditory nerve (AN) data tended to favour the beating of multiple closely spaced cochlear resonances and perhaps the existence of two distinct traveling waves. However, the results were not clear-cut, since inferring BM dynamics from AN recordings is problematic. So although our findings generally support the Lin & Guinan interpretation, there are many factors at play and establishing firm links to their work is difficult.

To explain waxing and waning seen in the cases examined here, it has been assumed that the spectral widths of the gammatones are due to their brevity, not because their carrier frequencies are imprecise or wavering. On the SOG model, then, each gammatone is said to originate from the oscillation of a resonant element whose frequency is physically fixed, so if it could be made to ring for longer, its frequency would be the same as that found by the curve-fitting algorithm (in which the frequencies were specified to the nearest 10 Hz). In other words, there are no gammachirps in this picture. The SOG model requires that the phase of the frequencies found by curve fitting need to be stable over the entire length of the recorded signal—many milliseconds—for destructive interference to occur and regular waxing and waning to be produced.

### Glides

Together with waxing and waning, frequency glides—typically a steep initial rise in IF—are a consistent feature of cochlear impulse responses which any model of cochlear mechanics needs to accommodate. The explanation of glides has previously been given either in terms of dispersion of the cochlear traveling wave (Shera, 2001b) or in the build-up and decay of multiple micromechanical resonances (Lin & Guinan, 2000). The glide has generally been seen as a separate phenomenon to waxing and waning, but here the advantage of seeing both as manifestations of beating—in line with the Lin and Guinan perspective—is set out. It is therefore suggested that both phenomena could be the result of local activity in the cochlea, not global. The literature on glides is extensive (De Boer & Kruidenier, 1990; Møller & Nilsson, 1979; Nilsson & Møller, 1977; Robles, Rhode & Geisler, 1976; Wilson & Johnstone, 1975), but here the discussion is limited to aspects bearing on the issue of beating.

A generally unappreciated aspect of beating is that at instants of destructive interference there are upwards/downwards surges above the mean frequency, or downwards/upwards surges below the mean frequency, depending on the relative amplitude of each component (see Fig. A3 of Supplementary Material S1). So if there are two gammatones with different onset and decay rates, the direction of the surge at the instant of destructive interference will depend on the relative amplitude of each component, and this will change with time (since the envelopes rise and fall at different rates), allowing either an upwards- or downwards-pointing surge to occur depending on the relative frequencies and amplitudes of the beating components at that moment. This binary outcome was used to explain the IFs of the Shera and Cooper waveform (Case 2).

Thus, for the four gammatones isolated in Case 2 (shown in Fig. 6B), the theoretically computed IFs (by Hilbert transform) of their sum reproduce the IFs found in the actual waveform (Fig. 6D). The directions of each of the sequential glides in Fig. 4B can simply be predicted by taking into account the relative frequency of the dominant gammatone at each instant (see Fig. A3). Relative amplitudes and frequencies of putative gammatones might also be used to explain the directions of IF surges observed by De Boer & Nuttall (1997).

The relative amplitude factor can also be used to explain why, depending on CF, initial IF trajectories systematically change direction. Carney, McDuffy & Shektar (1999) reported that nerve fibre recordings from cats showed steep upwards trajectories for high frequency fibres and downwards trajectories for low frequency fibres, with a steady transition from one to the other. A similar pattern of a change in the direction of glides, depending on frequency, can be seen in Fig. 17 of Recio-Spinoso et al. (2005). This property is a challenge to explain using usual dispersion models (Guinan & Nam, 2018), but on the beating gammatone model the pattern results from a change in the mix of two underlying components: the higher frequency one dominates the impulse response for high CFs (giving an upwards-pointing surge), but for low CFs, below about 1 kHz in the case under consideration, the lower frequency component has the greater amplitude and gives a downwards-directed surge.

The regular appearance of upward frequency glides at the beginning of an impulse response suggests destructive interference at this point, implying that it is the second oscillator, like *m*_2_ in Fig. 16, which is being observed, and that it is in phase opposition at this instant to its unobserved companion (*m*_1_).

Again, the limitations mentioned earlier need to be kept in mind. In particular, it is impossible to be sure that all the gammatones are real, in the sense of each one arising from a single oscillating element, or whether together they are approximating another process that might be better described with a gammachirp, for example. Case 1 has shown that the greater the number of gammatones, the better the glide can be approximated, but this is understandable just in terms of the accuracy of the fit and relates again to the question of whether all the recovered gammatones can be considered ‘real’. Two gammatones by themselves appear to be unable to accurately explain the glide (Fig. 2), so perhaps dispersion and gammachirps still have an explanatory role to play. The last section of the ‘Discussion’ provides a broader perspective on this. The gammatone/gammachirp issue is left for further investigation.

A distinctive feature of some early papers (Nuttall & Fridberger, 2012; Recio et al., 1998; Ruggero & Rich, 1991a, 1991b), although not commented upon in their texts, is the appearance of a double-peaked spectrum, with a ratio between the peaks of about 1.1. De Boer & Nuttall (1997) found that the glide was present at stimulus intensities as low as 20 dB SPL where nonlinear effects would be minimal, and continued even after the envelope’s amplitude had begun to fall. The authors concluded that the glide is an essential property of the cochlea, which exists for all levels and for all CFs between at least 1.8 and 18 kHz. A later modelling paper (De Boer & Nuttall, 2000) revealed another remarkable property: zero-crossing invariance. As the intensity of a stimulus rises, the times at which impulse response waveforms cross the time-axis remain more or less fixed (Recio & Rhode, 2000; Shera, 2001c). Our analyses clearly revealed frequency glides (Figs. 6D and 7C), and in general we found that glide profiles (IF vs. time) were insensitive to intensity, as expected from zero-crossing invariance.

Because beating is a linear phenomenon, it follows that zero crossings of a beating waveform will normally be intensity invariant, and so the beating model explains zero-crossing invariance if the contributing sources are linear and derive from two fixed-frequency resonators. Of course, if the sources are nonlinear, deviations in zero crossings are expected (Recio-Spinoso, Narayan & Ruggero, 2009).

### Gammatones and two coupled oscillators

Our analysis has shown that cochlear impulse responses can be analysed into sets of discrete gammatones, and earlier it was suggested that a locally based resonance picture may be of value in providing an insight into the underlying mechanics. In this section, we look at the micromechanics more closely and pose the question of whether the two degrees of freedom of the coupled TM/BM system may be evident in broader-scale features. That is, if the cochlea contained isolated pairs of elastically coupled oscillators having two degrees of freedom, is it possible that the gammatones which form the ‘atoms’ of this system could remain as components of BM impulse responses?

The modelling of two elastically coupled masses (Case 6)—the simplest possible 2-DOF model—indirectly supports the reality of the gammatones. Case 6 showed that the impulse response of such a system gives rise to a waveform of the putative BM which closely resembles the sum of two second-order gammatones. The shape of the calculated response—similar to the beating of two gammatones—supports the idea that, within a local oscillator model of the cochlea, this waveform is preserved in the impulse response.

The first to introduce two degrees of freedom were Neely & Kim (1986) who represented the micromechanics of the cochlea with two masses: one the BM and the other, coupled to it, the TM. The model used an active pressure term, presumed to derive from the outer hair cells, to drive the BM and produce sharp tuning and high sensitivity; essentially, the partition was driven not by sound pressure alone but by the cochlear amplifier as well. The active elements provided negative damping, which allowed the system to have considerable power gain, sharp tuning, and—if given excessive gain—spontaneous activity. The authors showed that the 2-DOF model could provide a good match to tuning characteristics seen experimentally, and it prompted similar work based on coupled BM/TM systems (Liu & Neely, 2009; Meaud & Grosh, 2010; Richardson, Lukashkin & Russell, 2008; Sellon et al., 2015).

The suggestion that the first oscillator might be the TM and the second might be the BM is consistent with the twin-engine model of Aranyosi (2006) where this same assignment is made. Aranyosi, who compares the outputs of his model with BM impulse responses, makes both oscillators nonlinear and active—hence the twin-engine name—but for simplicity the treatment here considered the oscillators as linear and passive. Our results are nevertheless broadly consistent with his findings.

Recent non-invasive work has found that the TM is tuned slightly higher than the BM (Lee et al., 2015). This work, the basis of Case 5, found that, at the same longitudinal location, the TM of the mouse cochlea is tuned about 0.3 kHz higher than the BM, a difference producing a TM/BM frequency ratio of about 1.04 and which is expected to generate beating in impulse responses. A related observation from the guinea pig (Zheng et al., 2011) also suggests a frequency gap. In this case, the authors measured local electrical potentials in the organ of Corti at the same time as they measured its motion in response to sound. After the sound was switched off, waveforms recorded from the same location (their Fig. 5C) had different frequencies of decay: electrical activity showed up at 19.0 kHz while mechanical motion occurred at 16.4 kHz (a ratio of 1.16) and an initial phase difference of 180° was evident. The TM appears to be highly charged and piezoelectric (Ghaffari et al., 2013), which might connect the electrical waveform to the TM, but in any case the two distinct frequencies suggest separate sources, and Ghaffari et al. speculate that the TM and BM undergo independent vibration (ibid., p. 1631).

### Open questions

Although the foregoing provides possible contexts for how our findings might be explained, in the end definitive answers are elusive and there are multiple open questions, some of which are set out below.

There is a leap in moving from the most basic 2-DOF model and its gammatone-like waveform to more complex cases involving the real cochlea, with no guarantee that in a distributed and coupled cochlea a resonant element will still give rise to a gammatone. Other sorts of waveforms, such as gammachirps, cannot be ruled out (Irino & Patterson (1997); Meaud & Lemons (2015)). Where on the BM might all the multiple gammatones come from? Lee et al. (2016) measured simultaneous vibrations of not just the BM and TM but also the RL, and these vibrations appear more complex than the energy exchange of just two masses.

Another unresolved issue relates to the order of the gammatones. As modelling of two coupled masses showed, the basic 2-DOF model generates a waveform for the second mass which resembles a gammatone of order 2 (Case 6). However, we have used gammatones of order 3 or 4 in most of our fits. Some initial testing indicated that using gammatones of order 2–5 did not appreciably change the quality of fits, but this aspect requires verification.

Despite these shortcomings, there are a number of connections which may help to bridge the gap. One interesting symmetry is a similarity between the standard traveling wave as formulated by Békésy and the classical resonance model suggested by Helmholtz. Thus Bell (2012) examined the behaviour of a graded bank of uncoupled resonators which were independently and simultaneously excited. The tuning of the resonators was made to match that of the human cochlea, and the result was that an apparent traveling wave formed which had a phase velocity comparable to observed traveling wave velocities. In related work, Bell and Wit examined the behaviour of the resonance-based vibrating reed frequency meter (Bell & Wit, 2015) and found that when the reeds were elastically coupled this model produced complex dynamics such as traveling waves, phase plateaus, primary and secondary peaks, and a remarkable pattern of frequency corrugations in which neighbouring reeds clustered together at small frequency ratios. The impulse response of some of the reeds also showed waxing and waning. The differences between traveling wave models and local resonance models may therefore be less marked than often portrayed, and the gap would be further bridged if the introduction of cochlear roughness was shown to be equivalent to introducing echoes, as the analysis by Papoulis (1962) implies. The MIR model relies on roughness to generate internally reflected echoes (Meaud & Lemons, 2015; Li & Grosh, 2016), but any regular amplitude modulation of a signal—which might arise in a finite-element model due to nonlinearities introduced into the transduction channels—is theoretically equivalent to a series of time delays or echoes (Ch. 6–2 of Papoulis (1962)).

In a related fashion, it should be noted that there seems to be an equivalence between time delays and frequency differences. The way Zweig expresses it is that in an isolated cochlear oscillator a time delay feedback force can be replaced with an instantaneous nonlocal spatial interaction (Zweig, 2003, p. 323) and that nonlocal temporal interactions in a long-wavelength model behave like instantaneous interactions in a short-wavelength model (Zweig, 2015, p. 1102).

These possible connections need more detailed investigation, but a wider perspective might be that the traveling wave and resonance models might be regarded as complementary ways of describing the same thing. It may be preferable to adopt one particular viewpoint or the other depending on the phenomenon under investigation. In terms of the multiple resonances observed in the cochlea, the traveling wave picture runs into a number of anomalous behaviours (Guinan & Nam, 2018) and so the stationary resonance picture seems to come with certain advantages. If the traveling wave and resonance pictures were considered complementary, then the longstanding dichotomy between Helmholtz and Békésy might be brought closer together. Nevertheless, such an enterprise remains a challenge. Even if the underlying mathematics is similar, the phenomena themselves may still be different, particularly in terms of how the cochlear fluids and structures are coupled. The frequency of the oscillators will depend on fluid properties (Lighthill, 1981), and the way the fluid moves will play a role in determining the impulse response. Finally, Helmholtz’s resonant strings cannot be totally independent since, to some degree, they must interact via the cochlear fluid (Duifhuis, 2012, p. 181).

So although major questions remain, the regularity of the SOG fitting process provides an intriguing new perspective on cochlear mechanics, suggesting that the cochlea might contain a set of closely spaced resonant elements.

## Conclusion

This investigation of BM impulse responses demonstrates that, to a good approximation, they can be decomposed into at least two and up to six fixed-frequency gammatones each equally spaced in frequency by a ratio of about 1.1. If the impulses are prolonged, interaction of the two gammatones becomes evident as waxing and waning, which can be interpreted as a manifestation of beating. In turn, the destructive interference inherent to beating appears to give rise to a distinctive pattern of frequency glides, contributing to systematic upward and downward sweeps in frequency, most commonly seen as an initial upward glide.

For many years, the explanation for frequency glides in impulse responses has been that there is some dispersive process at work in the cochlea (Meaud & Lemons, 2015). Through dispersive traveling waves, it is thought that wavefronts come together to make the IF rise—as if rotating the tuning knob on a parametric oscillator. The results of the current work offer a different way of describing the phenomenon in terms of a set of local oscillators closely spaced in frequency. At its simplest, each lumped set of oscillators might be considered to act like a single oscillator, perhaps in a similar way to which clusters in a chain of coupled oscillators can behave like a single oscillator (Wit & Bell, 2017). Whether the local oscillators actually exist and gammatones are at work—the system is stationary—or whether there is dispersive wave behaviour involving a gammachirp or other nonstationary waveform, is a matter left for further research. If the SOG method is valid and genuinely picks out local cochlear resonances, then perhaps it might also be usefully applied to click-evoked otoacoustic emissions as well (Wit, Van Dijk & Avan, 1994).

In summary, the SOG approach has the potential to open new doors in understanding the cochlea and its subtle dynamics, but there are a number of major gaps which first need to be filled.

## Supplemental Information

10.7717/peerj.6016/supp-1 Supplemental Information 1 Example of a Mathematica notebook illustrating how gammatones were fitted to an impulse response. The example is the chinchilla impulse response recorded by Shera & Cooper (2013), the basis of Fig. 6.

10.7717/peerj.6016/supp-2 Supplemental Information 2 Properties of beating. Beating describes the fluctuations in amplitude which occur when two tones of closely spaced frequency combine. This text summarises distinctive properties which arise from beating, notably glides in instantaneous frequency.

10.7717/peerj.6016/supp-3 Supplemental Information 3 Impulse response of two coupled harmonic oscillators. This section derives the impulse response of two elastically coupled oscillators. The impulse response is shown to exhibit a waveform which waxes and wanes. It is shown that the waveform can be closely fitted with two component gammatones. That is, waxing and waning can be interpreted as the beating of two underlying gammatones.

## Additional Information and Declarations

### Data Availability

The following information was supplied regarding data availability: The raw data exists as six separate files. Each is an impulse response corresponding to CASES 1–5 as discussed in the text (there are two files for CASE 4 corresponding to the masked and unmasked conditions). In each case, the first column is time, and the second column is displacement. An example of code used in fitting gammatones to impulse responses is provided as a Mathematica notebook (see Supplementary Information). It describes the steps used in creating Fig. 6. The research in this article did not generate any new data. The raw data we used came from the researchers we have cited who have previously published it in journals and have kindly made it available to us. The names and contact details of the people from whom raw data can be requested are:

1. Case 1, from Ren, He & Barr-Gillespie (2016). Data supplied by Tianying Ren, Oregon Hearing Research Center, Oregon Health & Science University, Portland, Oregon 97239, USA. Email: rent@ohsu.edu.
2. Case 2, from Shera & Cooper (2013). Data supplied by Christopher Shera, Otolaryngology HRA 326–1640, Marengo St, Health Sciences Campus, Keck School of Medicine, University of Southern California, Los Angeles, CA 90033, USA. Email: christopher.shera@usc.edu.
3. Case 3, from Recio & Rhode (2000). Data supplied by Alberto Recio-Spinoso, Universidad de Castilla–La Mancha, Albacete, Spain. Email: alberto.recio@uclm.es.
4. Case 4, from Recio-Spinoso & Cooper (2013). Data supplied by Alberto Recio-Spinoso (as per Case 3).
5. Case 5, from Elliott, Ni & Sun (2017). Data supplied by Guangjian Ni, Institute of Sound and Vibration Research, University of Southampton, Southampton SO17 1BJ, UK. Email: niguangjian@gmail.com.
