# Supplementary material for: Cochlear impulse responses resolved into sets of gammatones: the case for beating of closely spaced local resonances
Source: PeerJ. 2018 Nov 27;6:e6016. doi: 10.7717/peerj.6016 (PMC6266938; doi:10.7717/peerj.6016)
Supplement: Supplemental Information 1 — The example is the chinchilla impulse response recorded by Shera & Cooper (2013), the basis of Fig. 6. [file peerj-06-6016-s001.pdf]

This is the Mathematica notebook to calculate the figures in the panels of Figure 6.

```
In[1]:= SetDirectory["desktop/shera2018"]
Out[1]= /Users/hpwit/Desktop/Shera2018
```

Importing the LPHP filtered signal:

```
In[2]:= sigfilt = Import["sigHPLPfil.txt", "Table"];
In[3]:= ListLinePlot[sigfilt, PlotRange → All, AspectRatio → 0.3,
         PlotStyle → Orange, AxesLabel → {"time (ms)", "displacement (nm)"}]
```

Out[3]=

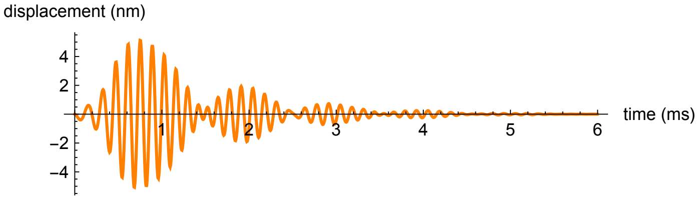

Fitting the filtered signal with the sum of 4 third order gammatones (starting values were found with Abscissa):

```
In[4]:= n = 2;
In[5]:= g1[t_] := α1 * t^n * Exp[-β1 * t] * Cos[2 * Pi * ξ1 * t + φ1];
In[6]:= g2[t_] := α2 * t^n * Exp[-β2 * t] * Cos[2 * Pi * ξ2 * t + φ2];
In[7]:= g3[t_] := α3 * t^n * Exp[-β3 * t] * Cos[2 * Pi * ξ3 * t + φ3];
In[8]:= g4[t_] := α4 * t^n * Exp[-β4 * t] * Cos[2 * Pi * ξ4 * t + φ4];
In[9]:= g[t_] := g1[t] + g2[t] + g3[t] + g4[t];
In[10]:= fit = FindFit[sigfilt, g[t], {{α1, 141.21}, {β1, 5.7647}, {ξ1, 6.7}, {φ1, 0.0},
          {α2, 9.662}, {β2, 1.827}, {ξ2, 6.966}, {φ2, 4.738}, {α3, 62.56}, {β3, 3.449},
          {ξ3, 7.583}, {φ3, 2.0}, {α4, 4.603}, {β4, 1.577}, {ξ4, 7.94}, {φ4, -1.14}}, t];
```

The fitted parameters $\alpha1$, $\beta1$, $\zeta1$, $\varphi1$ etc.:

```
In[11]:= p = Table[fit[[i]][[2]], {i, 1, 16}]
Out[11]= {141.199, 5.76476, 6.76142, -0.128393, 9.66252, 1.82743, 6.96607, 4.73775,
          62.5588, 3.4491, 7.58305, 1.99985, 4.60286, 1.57738, 7.93998, -1.13988}
```

Inserting the parameters in the formulas for the gammatones and their sum:

```
In[12]:= gam1[t_] := p[[1]] * t^n * Exp[-p[[2]] * t] * Cos[2 * Pi * p[[3]] * t + p[[4]]];
In[13]:= gam2[t_] := p[[5]] * t^n * Exp[-p[[6]] * t] * Cos[2 * Pi * p[[7]] * t + p[[8]]];
In[14]:= gam3[t_] := p[[9]] * t^n * Exp[-p[[10]] * t] * Cos[2 * Pi * p[[11]] * t + p[[12]]];
In[15]:= gam4[t_] := p[[13]] * t^n * Exp[-p[[14]] * t] * Cos[2 * Pi * p[[15]] * t + p[[16]]];
In[16]:= gam[t_] := gam1[t] + gam2[t] + gam3[t] + gam4[t];
```

Making a table for the sum, with time-axis:

```
In[17]:= gamtab = Table[gam[t], {t, 0, 6 - 0.005, 0.005}];
In[18]:= time = Table[t, {t, 0, 6 - 0.005, 0.005}];
In[19]:= gamtabt = Transpose[{time, gamtab}];
```

Calculating the table for the difference between the filtered signal and the fit:

```
In[20]:= sigfil = Transpose[sigfilt][[2]];

In[21]:= difsig = sigfil - gamtab;

In[22]:= dift = Transpose[{time, difsig - 6.0}];
```

Plotting the filtered signal, the fit and the difference between the two:

```
In[23]:= plot1 = ListLinePlot[{sigfilt, gamtabt, dift}, PlotRange → {-7.5, 6},
           AspectRatio → 0.5, AxesOrigin → {0, -7.5}, ImageSize → 450,
           PlotStyle → {{Orange, Thickness[0.007]}, {Blue, Thickness[0.004],
               Dashing[{0.005, 0.009}]}, {Darker[Cyan], Thickness[0.005]}},
           AxesStyle → Directive[{Black, Thickness[0.004], 15}],
           GridLines → {{1.48, 2.51, 3.54, 4.60}, None},
           GridLinesStyle → Directive[Black, Dashed, Thickness[0.003]]]
```

Out[23]=

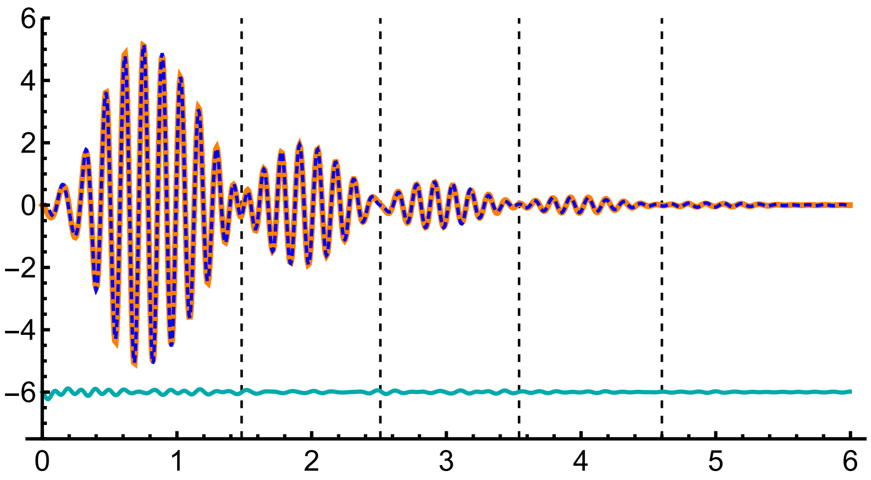

RMS-value of signal and difference:

```
In[24]:= ms = Sqrt[Mean[sigfil^2]]

Out[24]= 1.25166

In[25]:= md = Sqrt[Mean[difsig^2]]

Out[25]= 0.0354099
```

Their ratio:

```
In[26]:= md / ms

Out[26]= 0.0282904
```

Plotting the fitted gammatones:

```
In[27]:= gam1tab = Table[gam1[t], {t, 0, 6 - 0.005, 0.005}];

In[28]:= gam1tabt = Transpose[{time, gam1tab + 7}];

In[29]:= gam2tab = Table[gam2[t], {t, 0, 6 - 0.005, 0.005}];

In[30]:= gam2tabt = Transpose[{time, gam2tab + 3.5}];

In[31]:= gam3tab = Table[gam3[t], {t, 0, 6 - 0.005, 0.005}];

In[32]:= gam3tabt = Transpose[{time, gam3tab - 1}];
```

In[33]:=
```
gam4tab = Table[gam4[t], {t, 0, 6 - 0.005, 0.005}];
```

In[34]:=
```
gam4tabt = Transpose[{time, gam4tab - 5}];
```

In[35]:=
```
plot2 = ListLinePlot[{gam1tabt, gam2tabt, gam3tabt, gam4tabt},
  PlotRange → {-7, 10}, AspectRatio → 0.5, AxesOrigin → {0, -7}, ImageSize → 450,
  PlotStyle → {{Purple, Thickness[0.004]}, {Darker[Blue], Thickness[0.004]},
    {Brown, Thickness[0.004]}, {Green, Thickness[0.004]}},
  AxesStyle → Directive[{Black, Thickness[0.004], 15}],
  GridLines → {{1.48, 2.51, 3.54, 4.60}, None},
  GridLinesStyle → Directive[Black, Dashed, Thickness[0.003]]]
```

Out[35]=
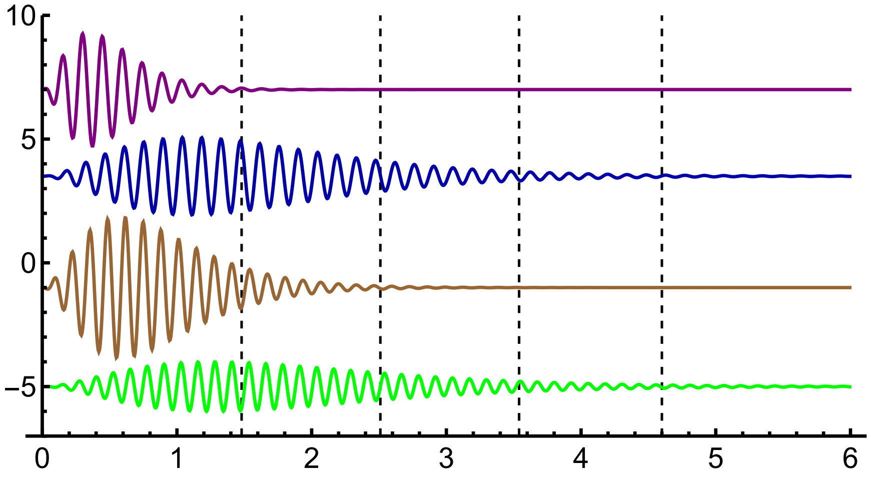

Zero-padding the filtered signal to obtain a smooth frequency spectrum:

In[36]:=
```
sigfz = Join[Table[0.0, {i, 1, 4400}], sigfil, Table[0.0, {i, 1, 4400}]];
```

Fourier-transform of the signal:

In[37]:=
```
fsigfz = Fourier[sigfz];
```

Its absolute value:

In[38]:=
```
afsfz = Abs[fsigfz];
```

Plotted with the right frequency-axis:

In[39]:=
```
freqlong = Table[0.02 * i, {i, 0, 9999}];
```

In[40]:=
```
fafsfz = Transpose[{freqlong, afsfz / Max[afsfz]}];
```

In[41]:=
```
ListLinePlot[Take[fafsfz, {100, 600}],
 PlotRange → All, AxesLabel → {"frequency (kHz)", "amplitude"},
 AspectRatio → 0.3, PlotStyle → Orange]
```

Out[41]=
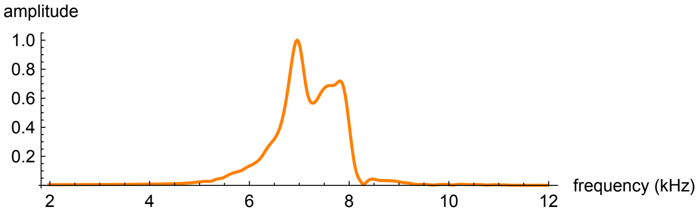

The same procedure for the gammatones:

In[42]:=
```
g1z = Join[Table[0.0, {i, 1, 4400}], gam1tab, Table[0.0, {i, 1, 4400}]];
```

```
In[43]:= fg1z = Fourier[g1z];

In[44]:= afg1 = Abs[fg1z] / Max[afsfz];

In[45]:= fafg1 = Transpose[{freqlong, afg1}];

In[46]:= ListLinePlot[Take[fafg1, {100, 600}], PlotRange → All,
          AxesLabel → {"frequency (kHz)", "amplitude"},
          AspectRatio → 0.3, PlotStyle → Purple]
```

Out[46]=

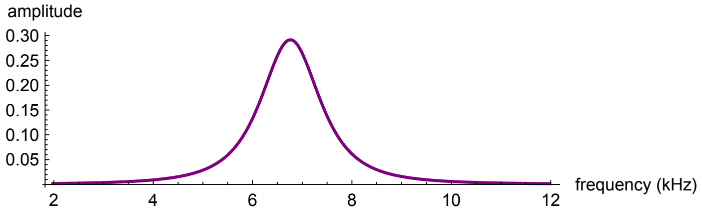


```
In[47]:= g2z = Join[Table[0.0, {i, 1, 4400}], gam2tab, Table[0.0, {i, 1, 4400}]];

In[48]:= fg2z = Fourier[g2z];

In[49]:= afg2 = Abs[fg2z] / Max[afsfz];

In[50]:= fafg2 = Transpose[{freqlong, afg2}];

In[51]:= ListLinePlot[Take[fafg2, {100, 600}], PlotRange → All,
          AxesLabel → {"frequency (kHz)", "amplitude"},
          AspectRatio → 0.3, PlotStyle → Darker[Blue]]
```

Out[51]=

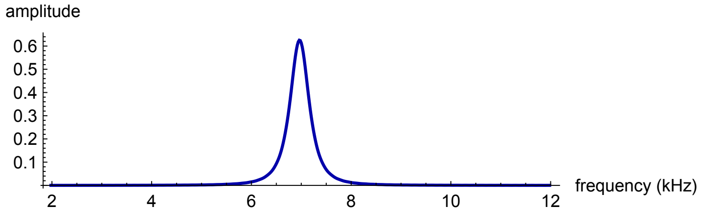


```
In[52]:= g3z = Join[Table[0.0, {i, 1, 4400}], gam3tab, Table[0.0, {i, 1, 4400}]];

In[53]:= fg3z = Fourier[g3z];

In[54]:= afg3 = Abs[fg3z] / Max[afsfz];

In[55]:= fafg3 = Transpose[{freqlong, afg3}];

In[56]:= ListLinePlot[Take[fafg3, {100, 600}], PlotRange → All,
          AxesLabel → {"frequency (kHz)", "amplitude"},
          AspectRatio → 0.3, PlotStyle → Brown]
```

Out[56]=

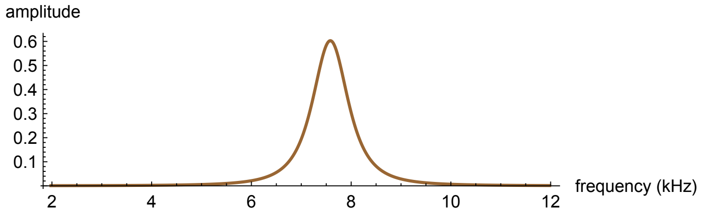


```
In[57]:= g4z = Join[Table[0.0, {i, 1, 4400}], gam4tab, Table[0.0, {i, 1, 4400}]];

In[58]:= fg4z = Fourier[g4z];
```

In[59]:=
```
afg4 = Abs[fg4z] / Max[afsfz];
```

In[60]:=
```
fafg4 = Transpose[{freqlong, afg4}];
```

In[61]:=
```
ListLinePlot[Take[fafg4, {100, 600}], PlotRange → All,
 AxesLabel → {"frequency (kHz)", "amplitude"},
 AspectRatio → 0.3, PlotStyle → Green]
```

Out[61]=

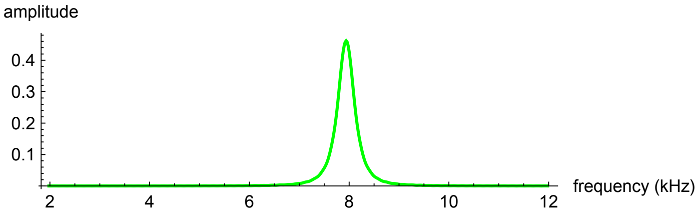

And for the amplitude spectrum of the sum of the gammatones:

In[62]:=
```
gsz = Join[Table[0.0, {i, 1, 4400}],
   gam1tab + gam2tab + gam3tab + gam4tab, Table[0.0, {i, 1, 4400}]];
```

In[63]:=
```
fgsz = Fourier[gsz];
```

In[64]:=
```
afgs = Abs[fgsz] / Max[afsfz];
```

In[65]:=
```
fafgs = Transpose[{freqlong, afgs}];
```

In[66]:=
```
ListLinePlot[Take[fafgs, {100, 600}], PlotRange → All,
 AxesLabel → {"frequency (kHz)", "amplitude"},
 AspectRatio → 0.3, PlotStyle → {Blue, Dashed}]
```

Out[66]=

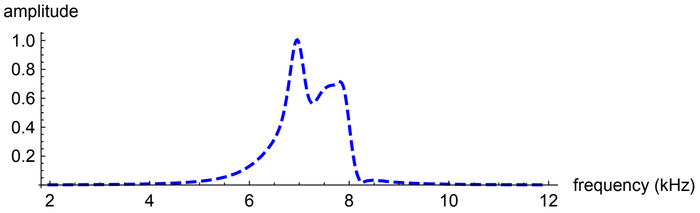

The amplitude spectra of the filtered signal, of the gammatones and of their sum:

In[67]:=
```
plot3 = ListLinePlot[{Take[fafg1, {250, 500}], Take[fafg2, {250, 500}],
    Take[fafg3, {250, 500}], Take[fafg4, {250, 500}], Take[fafsfz, {250, 500}],
    Take[fafgs, {250, 500}]}, PlotRange → All, AspectRatio → 0.5,
  PlotStyle → {Purple, Darker[Blue], Brown, Green, {Orange, Thickness[0.009]},
    {Blue, Thickness[0.005], Dashing[{0.01, 0.016}]}},
  AxesStyle → Directive[{Black, 15, Thickness[0.004]}], ImageSize → 400]
```

Out[67]=

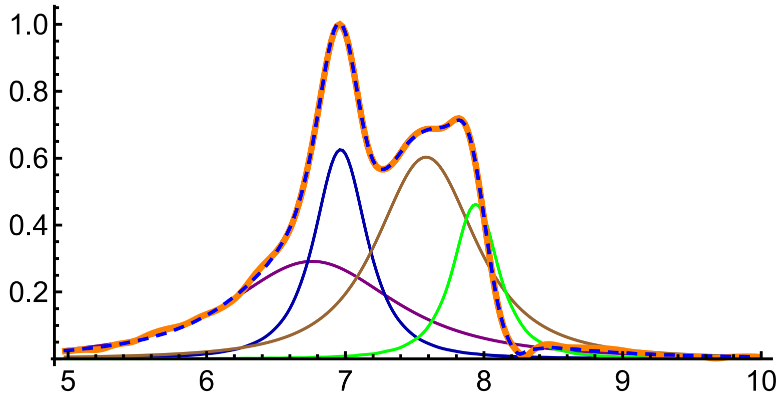

A detail showing the notch:

In[68]:=
```
f1 = 409; f2 = 434;
```

In[69]:=
```
plot3B = ListLinePlot[{Take[fafsfz, {f1, f2}], Take[fafgs, {f1, f2}]},
  PlotRange → All, AspectRatio → 0.7, PlotStyle →
   {{Orange, Thickness[0.02]}, {Blue, Thickness[0.02], Dashing[{0.01, 0.04}]}},
  AxesStyle → Directive[{Black, 15, Thickness[0.012]}],
  ImageSize → 150, Axes → {True, False}, Ticks →
   {{{8.2, "8.2", {0, -0.04}}, {8.3, "", {0, -0.025}}, {8.4, "8.4", {0, -0.04}},
     {8.5, "", {0, -0.025}}, {8.6, "8.6", {0, -0.04}}}, None}]
```

Out[69]=

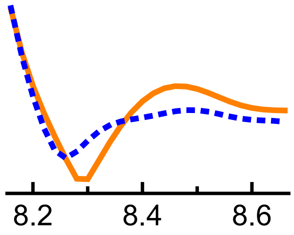

Calculating the phase of the filtered signal, using the Hilbert transform :

In[70]:=
```
fsf = Fourier[sigfil];
```

In[71]:=
```
hil = Join[Table[2.0, {i, 1, 600}], Table[0.0, {i, 1, 600}]];
```

In[72]:=
```
z = InverseFourier[hil * fsf];
```

In[73]:=
```
az = -Arg[z];
```

In[74]:=
```
ListPlot[az, Joined → True, PlotRange → All, AspectRatio → 0.2,
 AxesLabel → {"time  (5 μs units)", "phase φ (radians)"},
 PlotStyle → Darker[Green], ImageSize → 450]
```

Out[74]=

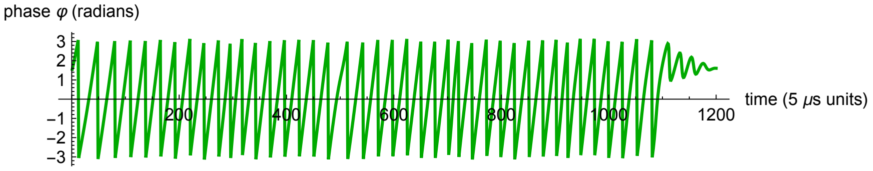

Calculating the slope dφ/dt of the phase and removing the 2π jumps:

In[75]:=
```
phislope = Table[0.0, {i, 1, 1200}];
```

In[76]:=
```
Do[phislope[[i]] = az[[i + 1]] - az[[i]], {i, 1, 1199}]
```

In[77]:=
```
Do[If[phislope[[i]] < -1.0,
   phislope[[i]] = (phislope[[i + 1]] + phislope[[i - 1]]) / 2.0,
   phislope[[i]] = phislope[[i]]], {i, 1, 1199}]
```

Calculating the instantaneous frequency IF:

In[78]:=
```
Δt = 6.0 / 1200.0;
```

In[79]:=
```
if = phislope / (2 * Pi * Δt);
```

Plotting IF with the right time axis:

In[80]:=
```
ift = Transpose[{Take[time, {1, Length[if]}], if}];
```

In[81]:=
```
ListPlot[ift, Joined → True, PlotRange → {0, 12}, AspectRatio → 0.3,
 PlotStyle → Orange, AxesLabel → {"time  (ms)", "IF  (kHz)"}]
```

Out[81]=

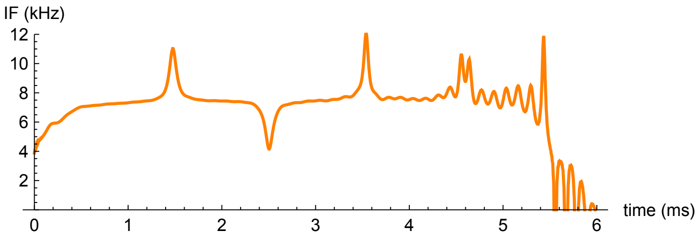

The same procedure for the sum of the gammatones:

In[82]:=
```
fgt = Fourier[gamtab];
```

In[83]:=
```
zg = InverseFourier[hil * fgt];
```

In[84]:=
```
azg = -Arg[zg];
```

In[85]:=
```
phislopeg = Table[0.0, {i, 1, 1200}];
```

In[86]:=
```
Do[phislopeg[[i]] = azg[[i + 1]] - azg[[i]], {i, 1, 1199}]
```

In[87]:=
```
Do[If[phislopeg[[i]] < -1.0,
   phislopeg[[i]] = (phislopeg[[i + 1]] + phislopeg[[i - 1]]) / 2.0,
   phislopeg[[i]] = phislopeg[[i]]], {i, 1, 1199}]
```

In[88]:=
```
ifg = phislopeg / (2 * Pi * Δt);
```

In[89]:=
```
ifgt = Transpose[{Take[time, {1, Length[ifg]}], ifg}];
```

In[90]:=
```
ListPlot[ifgt, Joined → True, PlotRange → {0, 14}, AspectRatio → 0.3,
  PlotStyle → {Blue, Dashed}, AxesLabel → {"time (ms)", "IF (kHz)"}]
```

Out[90]=

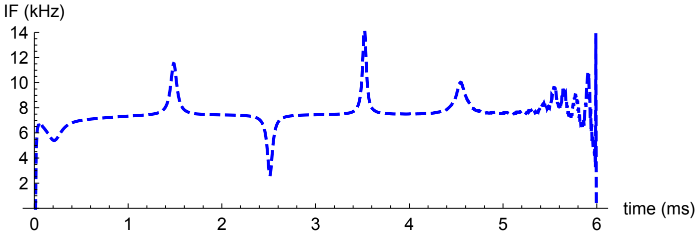

Making tables for horizontal lines indicating the frequencies of the gammatones:

In[91]:=
```
l1 = Transpose[{Take[time, {1, Length[if]}], Table[p[[3]], {i, 1, Length[if]}]}];
```

In[92]:=
```
l2 = Transpose[{Take[time, {1, Length[if]}], Table[p[[7]], {i, 1, Length[if]}]}];
```

In[93]:=
```
l3 = Transpose[{Take[time, {1, Length[if]}], Table[p[[11]], {i, 1, Length[if]}]}];
```

In[94]:=
```
l4 = Transpose[{Take[time, {1, Length[if]}], Table[p[[15]], {i, 1, Length[if]}]}];
```

Plotting the IFs for the filtered signal and the fitted sum of gammatones, together with the lines indicating the frequencies of the gammatones, for the time interval 0.2 - 3.7 ms :

In[95]:=
```
begin = 42;
```

In[96]:=
```
end = 740;
```

In[97]:=
```
plot4 = ListLinePlot[
  {Take[l1, {begin, end}], Take[l2, {begin, end}], Take[l3, {begin, end}],
   Take[l4, {begin, end}], Take[ift, {begin, end}], Take[ifgt, {begin, end}]},
  PlotRange → {{0, 4}, {5.3, 9}}, AspectRatio → 0.6, ImageSize → 400,
  AxesOrigin → {0, 5.3}, PlotStyle → {{Purple, Thickness[0.005]},
    {Darker[Blue], Thickness[0.005]}, {Brown, Thickness[0.006]},
    {Green, Thickness[0.006]}, {Orange, Thickness[0.008]},
    {Blue, Thickness[0.006], Dashing[{0.01, 0.016}]}},
  AxesStyle → Directive[{Black, Thickness[0.004], 18}],
  GridLines → {{1.48, 2.51, 3.54}, None},
  GridLinesStyle → Directive[Black, Dashed, Thickness[0.003]]]
```

Out[97]=

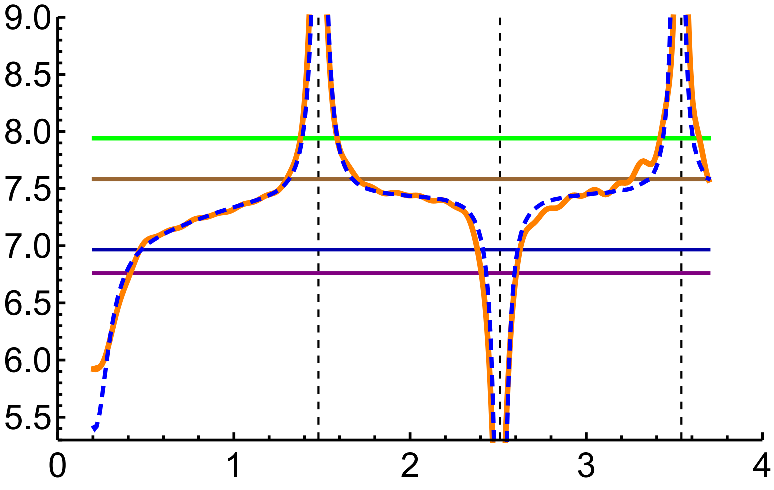

Plot1, plot2 etc. are exported to be used to compose Figure 6 with “Inkscape”.
