# Supplementary material for: Cochlear impulse responses resolved into sets of gammatones: the case for beating of closely spaced local resonances
Source: PeerJ. 2018 Nov 27;6:e6016. doi: 10.7717/peerj.6016 (PMC6266938; doi:10.7717/peerj.6016)
Supplement: Supplemental Information 2 — Beating describes the fluctuations in amplitude which occur when two tones of closely spaced frequency combine. This text summarises distinctive properties which arise from beating, notably glides in instantaneous frequency. [file peerj-06-6016-s002.pdf]

Supplementary Material S1

Properties of beating

Beating describes the fluctuations in amplitude which occur when two tones of closely spaced frequency combine, but behind this basic circumstance there are subtleties. This text summarises some distinctive properties which can also be found in basilar membrane click responses.

As described by Hartmann, beating is a phenomenon involving the linear combination of two tones of slightly different frequency (Ch. 17 of Hartmann (1998)). This linear beating needs to be distinguished from amplitude modulation (AM), which produces a similar waveform. Crucially, AM involves the *nonlinear* mixing of a carrier frequency and a modulating frequency, so a key difference is that the spectrum of a beating waveform shows only two frequencies, whereas AM shows three (a single carrier frequency plus two sidebands). Another important property of beating concerns the variations in instantaneous frequency which occur.

Consider two closely matched frequencies which interfere. If the component frequencies f_1 and f_2 have amplitudes a and b and angular frequencies ω_1 and ω_2, then the linear combination $x(t)$ is given by

$$x(t) = a \sin(\omega_1 t) + b \sin(\omega_2 t + \varphi) \ , \qquad \text{(A1)}$$

where t is time and φ is the relative phase. Two examples are shown in Figure A1 for frequencies of 1000 and 1100 Hz. At top, the amplitudes are equal; below, the amplitudes are in the ratio 2:1.

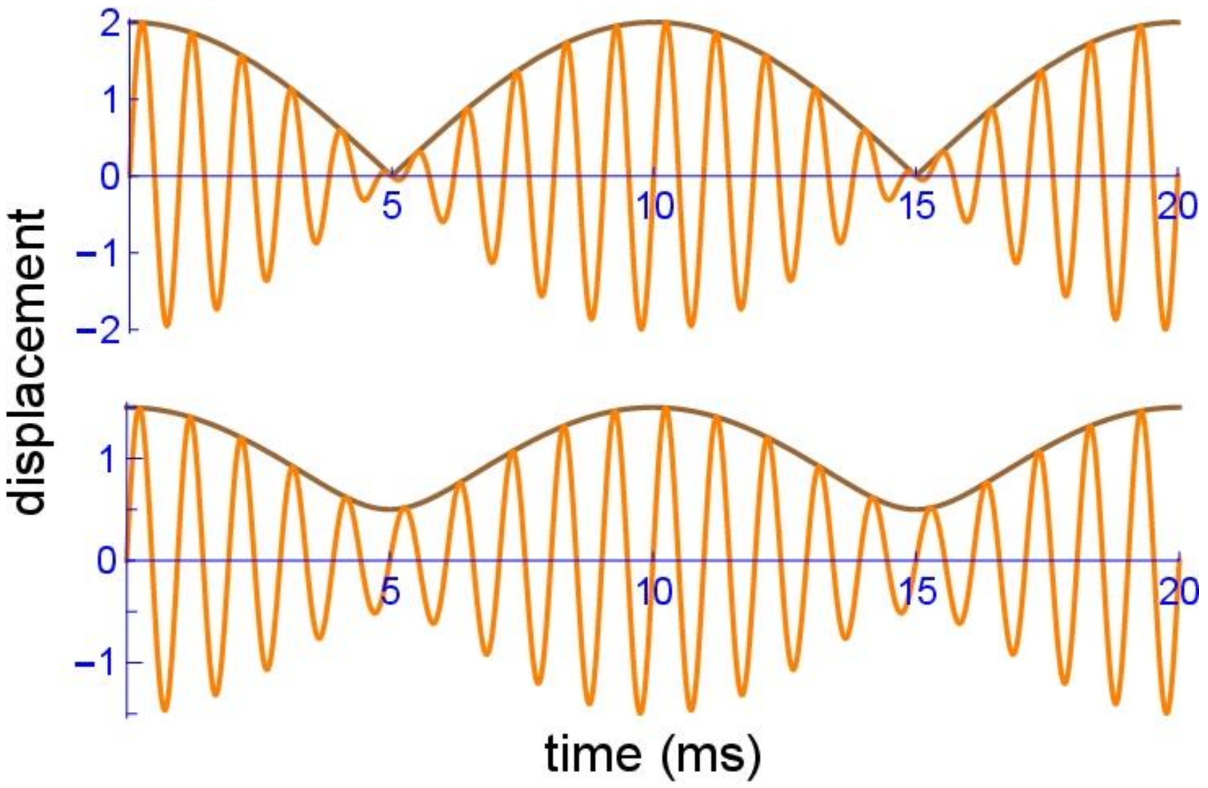

FIG. A1. Beating of two tones of frequencies 1000 and 1100 Hz, together with their envelopes. At top, the amplitudes of the two components, *a* and *b*, are equal ($a = b = 1$); below, $a = 1$ and $b = 0.5$ (ratio of 2:1).

From a spectral perspective, there are only two components, ω_1 and ω_2. However, listening to the waveform, the ear does not receive the impression of two distinct frequencies but instead that there is a single pure tone with a frequency half-way between the components and whose amplitude waxes and wanes ("beats") at the difference frequency $\omega_2 - \omega_1$. As Hartmann emphasises, although the difference frequency is heard, it does not – despite impressions – appear in the spectrum. This can be brought out by using a trigonometric identity and expressing Eq. (A1) as

$$x(t) = 2\cos\,[(\omega_1 t - \omega_2 t - \varphi)/2]\cdot\sin\,[(\omega_1 t + \omega_2 t + \varphi)/2]. \qquad \text{(A2)}$$

Defining the difference between ω_1 and ω_2 as $\Delta\omega$, and the average frequency $(\omega_1 + \omega_2)/2$ as $\overline{\omega}$, Eq. A2 becomes

$$x(t) = 2\cos\,((\Delta\omega/2)\,t - \varphi/2)\cdot\sin(\overline{\omega}\,t + \varphi/2) \qquad \text{(A3)}$$

in which the sine term represents rapid oscillation at the average frequency and the slowly oscillating cosine term represents the waxing and waning of amplitude. Although the cosine term oscillates at $(f_2 - f_1)/2$, there are $f_2 - f_1$ maxima per second (which is what is heard), half when the cosine term is +1 and half when it is –1. When the cosine wave crosses the time axis, the multiplier of the sine wave changes sign; in other words, the phase of the sine wave changes by 180°. This shows another important feature which distinguishes beats from amplitude modulation: the oscillating component rapidly changes phase by 180° between one burst and the next.

Consider Figure A2, which illustrates the beating of a pair of tones of 1000 and 1100 Hz, producing a waveform (orange) of periodicity 1050 Hz. For comparison, a sine wave of 1050 Hz is superimposed (blue), and it is evident that there is a sudden phase inversion from one beat to the next, meaning that each burst is a time-reversed copy of the preceding one and the waveform only repeats every two "bursts".

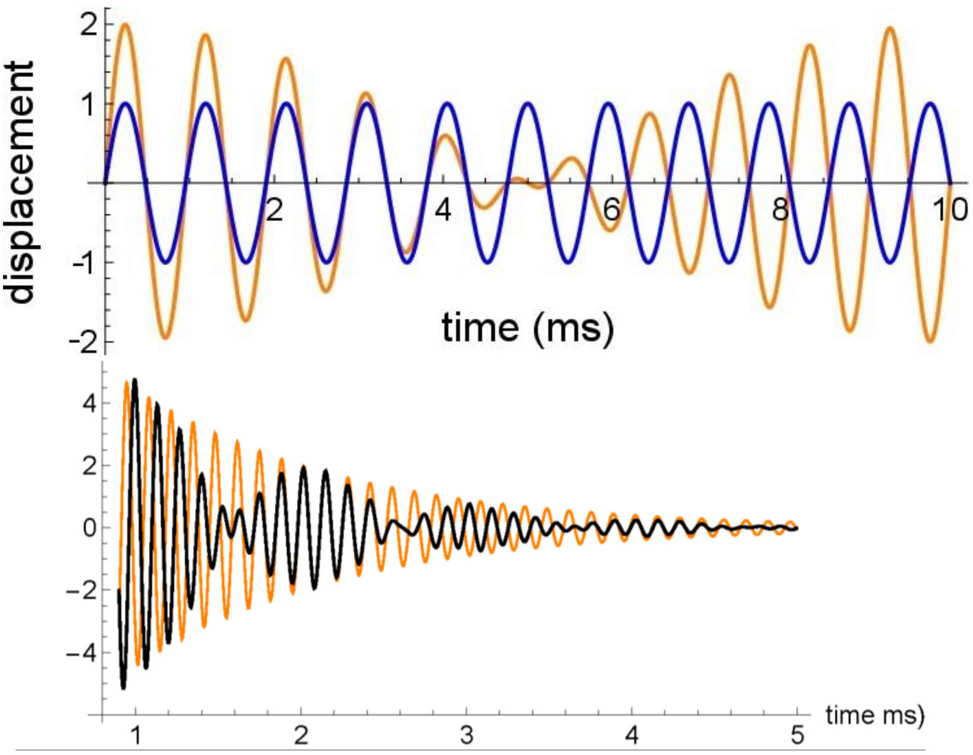

FIG. A2. (top) Waveform produced by the beating of two equal-amplitude tones of 1000 and 1100 Hz (orange) and a sine wave of frequency 1050 Hz (blue). Both have the same periodicity, but the phase changes suddenly by 180° at the cross-over. The rapid change in phase per unit time represents a high instantaneous frequency or frequency glide. Below is a portion of the impulse response from Case 2 (Shera and Cooper 2013), where a similar comparison with a 7.48 kHz decaying sine wave indicates that the signal also shows an alternating phase.

Hartmann proceeds to show another unexpected effect of this phase change: a periodic change in the instantaneous frequency (IF) of the beating tones. That is, the rate of change of phase varies over the beating cycle, and this produces a series of frequency glides. In the context of BM click responses, this is an important result.

Following Hartmann, one can derive the IF of two beating sines as:

$$\omega(t) = \overline{\omega} + (\Delta\omega/2)(b^2 - a^2) / [a^2 + b^2 + 2ab \cos(\Delta\omega t + \phi)], \qquad \text{(A4)}$$

which shows the regular variation of the average frequency. When the beating waveform shown at the bottom of Figure A1 is considered, its IF is shown as the blue curve in Figure A3.

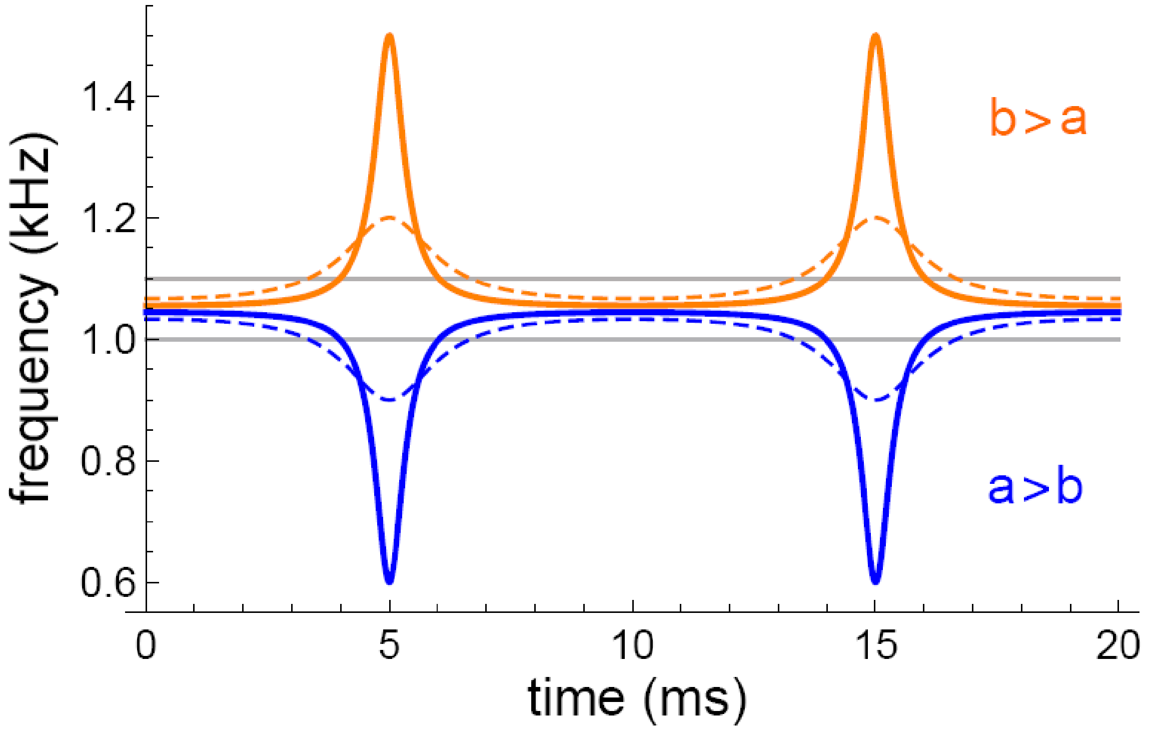

FIG. A3. Instantaneous frequencies (coloured lines) of two beating sines of 1000 and 1100 Hz (grey lines) for cases of different relative amplitude. The IFs of the beating waveforms go through surges in frequency at times of destructive interference, and it is the ratio of the amplitudes which determines the extent and direction of the surges. If the amplitude of the 1000 Hz tone is a and that of the 1100 Hz tone is b, then the *blue curves* illustrate the downward surges that occur when $a > b$ ($a = 1.25\,b$, solid blue curve; $a = 2\,b$, dashed blue curve). Conversely, if $b > a$, the IF surges are upward (*orange curves*, solid for $b = 1.25\,a$; dashed for $b = 2a$). In general, the closer the amplitudes, the narrower and more far-ranging are the surges.

The periodic changes in IF take the form of a rollercoaster of frequency surges. Moreover, the surge can be upwards or downwards depending on which component has the larger amplitude (as interchanging a and b in Eq. A4 makes clear). Figure A3 shows both downwardly and upwardly directed surges, with the IF dipping down when $a > b$ (blue curves), but rising upwards when $b > a$ (orange curves). If the amplitudes are nearly equal, the change in phase is almost instantaneous, and the frequency can rise from zero or descend from infinity depending on which component is larger. Interestingly, these changes in IF are audible, so that if certain parts of the waveform are edited out and the residual played to a listener, they will

report a frequency matching the weighted frequency of the residual (see Hartmann and its references).

The instantaneous frequency of any waveform, theoretical or measured, can be derived from the Hilbert transform of its 'analytic signal' (see Appendix C of de Boer & Nuttall (1997)). The other way of deriving the instantaneous frequency of a waveform is to calculate the inverse of the period between its zero crossings; both procedures usually give the same result (see footnote 14 of Shera (2001)).

There is also a useful relationship between the number of oscillations within a burst and the frequency ratio of the component tones. Equation 3 shows that the period of the fast oscillation is $2/(f_1 + f_2)$ seconds, and that there are n such periods within a beat envelope of $1/(f_2 - f_1)$ seconds. Therefore,

$$n \times [2/(f_1 + f_2)] = 1/(f_2 - f_1) \ ,$$

so that $n = (f_1 + f_2)/[2(f_2 - f_1)]$. (A5)

Define r as the ratio of f_2 and f_1, so that $f_2 = r\, f_1$. Substitution in Eq. A5 then implies that

$$n = (r + 1) \,/\, [2(r - 1)] \quad \text{or}$$

$$r = (2n + 1) \,/\, (2n - 1). \qquad \text{(A6)}$$

Equation A6 expresses the fact that the number of waves observable within a single beat reveals the ratio of the two component frequencies, and this simple relationship proves useful when inspecting cochlear waveforms. Thus, because the most common ratio between two gammatones is about 1.1, Eq. A6 explains why the number of cycles observed in later lobes of cochlear impulse responses tends to be roughly 10.

References

de Boer E, Nuttall AL. 1997. The mechanical waveform of the basilar membrane. I. Frequency modulations ("glides") in impulse responses and cross-correlation functions. *Journal of the Acoustical Society of America* 101:3583-3592 DOI 10.1121/1.418319.

Hartmann WM. 1998. *Signals, Sound, and Sensation*. New York: Springer.

Shera CA. 2001. Frequency glides in click responses of the basilar membrane and auditory nerve: their scaling behavior and origin in traveling-wave dispersion. *Journal of the Acoustical Society of America* 109:2023-2034 DOI 10.1121/1.1366372.
