# Supplementary material for: Cochlear impulse responses resolved into sets of gammatones: the case for beating of closely spaced local resonances
Source: PeerJ. 2018 Nov 27;6:e6016. doi: 10.7717/peerj.6016 (PMC6266938; doi:10.7717/peerj.6016)
Supplement: Supplemental Information 3 — This section derives the impulse response of two elastically coupled oscillators. The impulse response is shown to exhibit a waveform which waxes and wanes. It is shown that the waveform can be closely fitted with two component gammatones. That is, waxing and waning can be interpreted as the beating of two underlying gammatones. [file peerj-06-6016-s003.pdf]

Supplementary Material S2

Two coupled harmonic oscillators

This section derives the impulse response of two elastically coupled oscillators. The impulse response is shown to exhibit a waveform which waxes and wanes. It is shown that the waveform can be closely fitted with two component gammatones. That is, the waxing and waning can be interpreted as the beating of two underlying gammatones.

Consider two harmonic oscillators with the same natural frequency ω_0 and the same damping factor γ. The strength of the coupling force between the oscillators is proportional to the difference between the instantaneous displacements $x_1(t)$ and $x_2(t)$ – that is, the coupling is taken to be reactive with proportionality constant κ.

The set of equations to be solved is:

$$x_1'' + 2\gamma x_1' + \omega_0^2 x_1 = \kappa(x_2 - x_1)$$
$$x_2'' + 2\gamma x_2' + \omega_0^2 x_2 = \kappa(x_1 - x_2),$$

where x_1 is the oscillator receiving the impulse and x_2 is the oscillator elastically connected to it.

The initial conditions are chosen as $x_1(0) = x_2(0) = 0$ and $x_1'(0) = v_0$, $x_2'(0) = 0$.

By adding the two equations above, and with $y = x_1 + x_2$ we get:

$y'' + 2\gamma y' + \omega_0^2 y = 0$, with $y(0)=0$ and $y'(0) = v_0$.

Because this is the differential equation for a damped harmonic oscillator, we try as a solution: $\alpha e^{-\beta t} \sin(\omega t + \varphi)$. The initial conditions $y(0)=0$ and $y'(0) = v_0$ give $\varphi = 0$ and $\alpha = v_0/\omega$.

Substituting $y = \alpha e^{-\beta t} \sin\omega t$ in the differential equation for y and combining sine and cosine terms gives $\beta = \gamma$ and $\omega = (\omega_0^2 - \gamma^2)^{1/2}$.

So $x_1 + x_2 = \alpha e^{-\gamma t} \sin \omega t$, with $\alpha = v_0/\omega$ and $\omega = (\omega_0^2 - \gamma^2)^{1/2}$.

The consequence of this is that

$x_1 = \delta(v_0/\omega)e^{-\gamma t} \sin\omega t + f(t)$ and
$x_2 = (1- \delta)\,(v_0/\omega)\, e^{-\gamma t} \sin\omega t - f(t)$

where we suppose that $f(t) = \xi e^{-\gamma t} \sin(\chi t + \phi)$.

The initial conditions for x_1 and x_2 then yield $\phi = 0$ and $\xi = (1-\delta)v_0/\chi$.

As the next step, substitute

$x_1 = \delta(v_0/\omega)e^{-\gamma t} \sin\omega t + (1-\delta)\,(v_0/\chi)e^{-\gamma t} \sin\chi t$ and
$x_2 = (1-\delta)\,(v_0/\omega)e^{-\gamma t} \sin\omega t - (1-\delta)\,(v_0/\chi)\, e^{-\gamma t} \sin\chi t$

in the starting equations,

$x_1'' + 2\gamma x_1' + \omega_0^2 x_1 = \kappa(x_2 - x_1)$
$x_2'' + 2\gamma x_2' + \omega_0^2 x_2 = \kappa(x_1 - x_2)$.

After combining sine and cosine terms and simplifying, the result is $\delta = 0.5$ and $\chi = (\omega^2 + 2\kappa)^{1/2}$, giving the solutions for $x_1(t)$ and $x_2(t)$ as:

$x_1(t) = (v_0/2)\,\left(\frac{\sin\omega t}{\omega} + \frac{\sin\chi t}{\chi}\right)\, e^{-\gamma t}$
$x_2(t) = (v_0/2)\,\left(\frac{\sin\omega t}{\omega} - \frac{\sin\chi t}{\chi}\right)\, e^{-\gamma t}$.

As a check, if, as before, $\omega = (\omega_0^2 - \gamma^2)^{1/2}$ and $\chi = (\omega^2 + 2\kappa)^{1/2}$, then for $\kappa = 0$ (no coupling), $\chi = \omega$, giving $x_1(t) = (v_0/\omega)e^{-\gamma t} \sin\omega t$ and $x_2(t) = 0$.

$x_1(t)$ and $x_2(t)$ are the sum and the difference of the equations for a damped harmonic oscillator (or a gammatone of order 1), with angular frequencies ω and χ respectively.

Figure 16 of the main text shows the displacements x_1 and x_2 of two elastically coupled masses, m_1 and m_2, after m_1 receives an impulsive force giving it a velocity v_0. As pointed out in the main text, the motion of the two masses resembles beating, with the motion of m_2 similar to the impulse responses of the basilar membrane. This latter waveform can be approximated by the sum of two second-order gammatones (the theory above shows that the sum of two first-order gammatones – decaying sines – would provide an exact fit).

Tucker

Impulse responses of the BM appear to rise and then decay with a roughly exponential time-constant. An exponentially decaying sine wave is a gammatone of order 1. An exponentially decaying sine wave with sudden onset is the transient response of a single oscillator (see for example section 1.8 of Rossing & Fletcher (1995)).

The basic resonator, a single pole, generates the simplest possible gammatone of order 1 – an exponential decay – when stimulated by an impulse. Gammatones of order 3 to 5 are typically used in modelling. As described by Lyon, the poles do not have to be exactly coincident, but even if the poles do not coincide, the waveforms produced are still gammatone-like (p.173 of Lyon (2017)). As discussed by Lyon, the symmetry of a real gammatone filter depends on its associated zeros, which affect the shape of the low-frequency tail, and these can be judiciously added or removed (Lyon 2017, p.166). In cochlear terms, the zeros affect only the frequencies below CF. It is of interest that coincident-pole filters are well known in other fields (Lyon 2017, p.177), and it is known that fitting the impulse response of any system with a discrete number of gammatones gives an excellent approximation (Papoulis 1962), effectively representing the system as a cascade of identical one-pole filters.

One of the first to realise the connection between filter cascades and the Gamma function was Tucker (1946), who analysed the result of cascading parallel RLC resonators. He connected buffer amplifiers between the resonating stages and examined the result experimentally and theoretically. The result is elegant: if one drives a second-order filter (or

harmonic oscillator) with a gammatone of order *n*, the output will be almost identical to a gammatone of order $n + 1$.

The result can be confirmed using numerical methods. For example, if a gammatone of order 3 is used to drive a harmonic oscillator, the result is a waveform that can be accurately fitted with a gammatone of order 4.

References

Lyon RF. 2017. *Human and Machine Learning: Extracting meaning from sound*. Cambridge: Cambridge University Press. DOI 10.1017/9781139051699.

Papoulis A. 1962. *The Fourier Integral and Its Applications*. New York: McGraw-Hill.

Rossing TD, Fletcher NH. 1995. *Principles of Vibration and Sound*. New York: Springer.

Tucker DG. 1946. Transient response of tuned circuit cascades. *Wireless Engineer* 23:250-258
